# Photo-cross-linked and pH-Switchable Soft Polymer Nanocapsules from Polyglycidyl Ethers

**DOI:** 10.1021/acs.macromol.3c01698

**Published:** 2023-12-29

**Authors:** Stefan Engel, Pascal M. Jeschenko, Marcel van Dongen, Jonas C. Rose, Dominic Schäfer, Michael Bruns, Sonja Herres-Pawlis, Helmut Keul, Martin Möller

**Affiliations:** † Institute of Technical and Macromolecular Chemistry (ITMC), RWTH Aachen University, Worringerweg 2, D-52074 Aachen, Germany; ‡ DWI—Leibniz-Institute for Interactive Materials, Forckenbeckstraße 50, D-52074 Aachen, Germany; § Max Planck School Matter to Life, Jahnstraße 29, D-69120 Heidelberg, Germany; ∥ Institute of Inorganic Chemistry (IAC), RWTH Aachen University, Landoltweg 1, D-52074 Aachen, Germany; ⊥ Institute for Applied Materials and Karlsruhe Nano Micro Facility, Karlsruhe Institute of Technology, Hermann-von-Helmholtz-Platz 1, D-76344 Eggenstein-Leopoldshafen, Germany

## Abstract

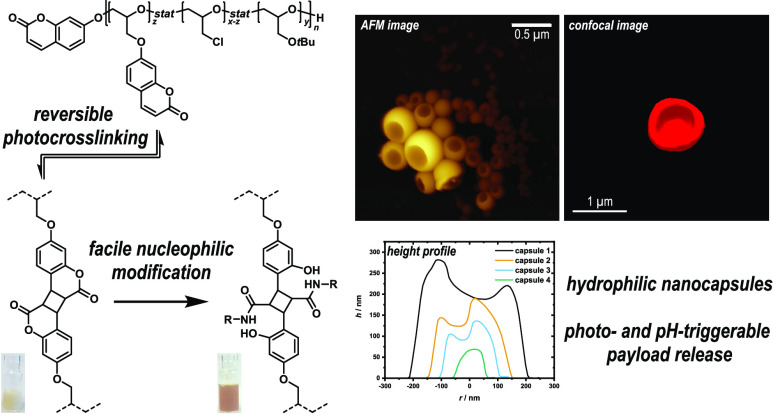

Soft polymer nanocapsules and microgels, which can adapt their shape and, at the same time, sequester and release molecular payloads in response to an external trigger, are a challenging complement to vesicular structures like polymersomes. In this work, we report the synthesis of such capsules by photo-cross-linking of coumarin-substituted polyglycidyl ethers, which we prepared by Williamson etherification of epichlorohydrin (ECH) repeating units with 7-hydroxycoumarin in copolymers with *tert*-butyl glycidyl ether (*t*BGE). To control capsule size, we employed the prepolymers in an o/w miniemulsion, where they formed a gel layer at the interface upon irradiation at 365 nm by [2π + 2π] photodimerization of the coumarin groups. Upon irradiation at 254 nm, the reaction could be reversed and the gel wall could be repeatedly disintegrated and rebuilt. We further demonstrated (i) reversible hydrophilization of the gels by hydrolysis of the lactone rings in coumarin dimers as a mechanism to manipulate the permeability of the capsules and (ii) binding functional molecules as amides. Thus, the presented nanogels are remarkably versatile and can be further used as a carrier system.

## Introduction

Macromolecular and colloidal carriers and substrates for functional molecules like drugs or catalysts offer a local chemical environment for tailoring their activity. Here, microgels combine aspects of both groups, macromolecules and colloids, such as having an open, penetrable architecture as well as constituting well-defined compartments.^1−3^ As a special feature, some hydrophilic microgels respond to variations in temperature or pH by swelling in water, i.e., they swell or shrink when the pH or the temperature changes due to alterations in the hydrophobic interaction.^4−6^ Such responsive or adaptive microgels gained interest for controlled uptake and release of drugs^7−9^ or as nanocompartments to regulate chemical and enzymatic activities.^10−14^

Lately, also light-sensitive microgels have been synthesized.^15^ Photosensitivity is not limited to exploit variations in the hydrophobic interaction but can extend responsiveness to nonaqueous dispersions. Furthermore, photoreactions can be controlled very selectively in manifold ways, i.e., by the choice of the wavelength, the intensity, and the polarization of the light, but also precisely in time and for the location. Photosensitive swelling has been reported by photoisomerization of substituents like azo groups or spiropyranes, which change their polarity upon photoisomerization, and also by light-induced splitting of a fraction of the cross-links.^16,17^ Particularly, photosensitive nitrobenzyl groups have been described either for protection of a cross-linking group (cageing) or for degradation of cross-links.^15,18−22^ For example, Landfester and Klinger prepared photodegradable PMMA microgels using bis(methacryloyl) cross-linkers with *o*-nitrobenzyl groups, which could be selectively addressed by adjusting the wavelength and irradiation time.^23^ Reversibility of cross-linking upon irradiation with ultraviolet (UV) light has been realized by cinnamates, chalcones, and coumarins. Upon irradiation at wavelengths of 320 to 370 nm, these compounds form dimers by [2π + 2π] cycloaddition, which can be cleaved by irradiation at a wavelength of <300 nm.^24−27^ The latter examples exploit a highly specific cross-linking reaction, [2π + 2π] cycloaddition, which barely interferes with the chemical functionality of biomolecules and which works in aqueous as well as in hydrophobic solvents. Lepage and Zhao described the reversible stabilization of polymer micelles, and nano- and microgels through cross-linking by photodimerization of coumarin groups attached to methyl methacrylates for entrapping functional molecules, which can be released on demand.^28−30^ Coumarin and chalcone dimers can be cleaved by two-photon adsorption using visible light, as demonstrated by Hampp et al. This way, these linker systems become applicable in UV absorbing material, damage by UV light is avoided, and the photoreactions become even more suitable in combination with biological compounds and living cells in particular.^31−33^

In this report, we focus on photosensitive microgels based on coumarin-functionalized poly(*tert*-butyl glycidyl ether)s as a prepolymer. These are hydrophobic prepolymers, which can be transformed to become hydrophilic by acidic hydrolysis. In order to obtain well-defined and uniform-sized microgels, we dissolved the prepolymers in hydrophobic solvents and dispersed this solution in a miniemulsion, which subsequently was irradiated for cross-linking. Not originally intended, we did not obtain spherical gel particles but hollow gel capsules corresponding to the original droplets as a template. These gel capsules are a new kind of nanogel capsule with an exterior shell that can be modified for hydrophilicity, by further substitution, and light-triggered degradation. We describe the synthesis of linear poly(*tert*-butyl glycidyl ethers), which are partly substituted by 7-hydroxycoumarin, their reversible photo-cross-linking in a miniemulsion, exploit reversible hydrolysis of the δ-lactone rings for pH-sensitive switching, and, finally, we demonstrate the transformation of the valerolactone groups in the coumarin dimers by trifluoroethylamine and 2,2-dipyrazol-1-yl-ethanamine.^34−36^ The former has been chosen to demonstrate the amide formation and 2,2-dipyrazol-1-yl-ethanamine in order to introduce the bis(pyrazolyl) scorpionate as a versatile ligand.

## Results and Discussion

**Scheme 1:**
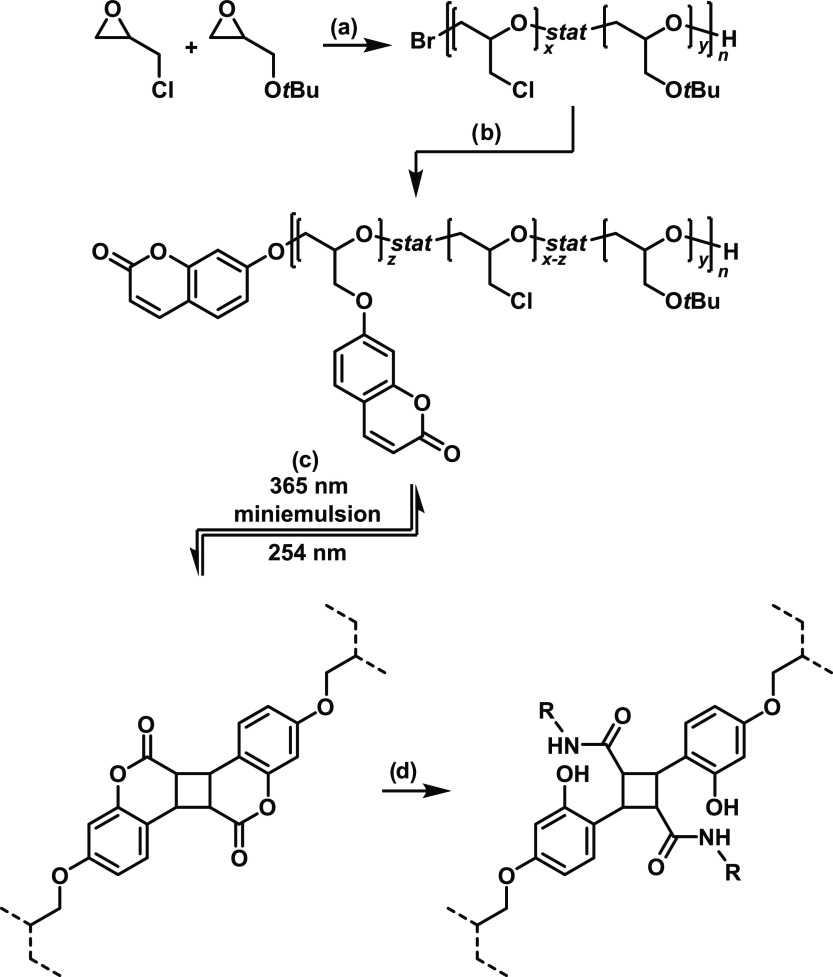
Preparation of Coumarin-cross-linked Polyglycidol Microgels and Post-cross-linking Functionalization (a) Monomer-activated anionic polymerization of ECH and *t*BGE Using *N*(*n*Bu)_4_Br and Al(*i*Bu)_3_ as Initiatiors; (b) Williamson etherification of ECH repeating units with 7-hydroxycoumarin using K_2_CO_3_ as the base, resulting in *p*(CumGE-*stat*-ECH-*stat*-*t*BGE); (c) reversible microgel synthesis in a miniemulsion by photochemical dimerization (λ = 365 nm) or cleavage (λ = 254 nm) of coumarin in *p*(CumGE-*stat*-ECH-*stat*-*t*BGE); and (d) microgel functionalization by lactone hydrolysis and amidation.

Scheme 1 describes the synthetic route to prepare the coumarin-substituted prepolymers, photo-cross-linking and aminolysis. Poly(ECH) synthesized via cationic ring-opening polymerization notoriously suffered from the formation of oligomeric rings.^37,38^ Ring formation should be avoided for the synthesis of the presented microgels. ECH consumed in the backbiting reaction is not available for further coumarin functionalization and the coexistence of rings and linear polymers could deteriorate the material’s mechanical properties. To circumvent this issue, we synthesized p(ECH*-stat-t*BGE) copolymers by a monomer-activated anionic ring-opening polymerization mechanism, using N(Bu)_4_Br and Al(*i*Bu)_3_ as the initiator and activator, respectively.^39,40^ No backbiting reactions with this initiation system have been reported in the literature.^41^ The reactivity of active alkoxide chain ends is significantly reduced by complexation of Al(*i*Bu)_3_. Propagation can only occur through nucleophilic addition to epoxides, which have been activated by complexation with additional Al(*i*Bu)_3_, increasing the selectivity of the reaction and reducing the risk of addition to other electrophilic functional groups, such as methylene chloride of ECH. Thus, only linear polymers are obtained. Substitution of chlorine atoms in p(ECH-*stat-t*BGE) polymers by 7-hydroxycoumarin resulted in the photosensitive prepolymers. After photodimerization, the [2π + 2π] adducts were reacted with primary amines to yield stable amides and to introduce further chemical functionalities. Before amidation, gelation can be reversed by proper choice of the wavelength and also the hydrolysis of the valerolactone rings is reversible by choice of the pH value. The hydrophilicity of the polymers and microgels can potentially be further controlled through acidic hydrolysis of the *t*BGE groups and the ratio of ECH to *t*BGE.^42−44^Table 1 summarizes molecular weights and compositions of the thus prepared copolymers.

**Table 1:** Monomer Ratio, Molecular Weight, and Yield of *p*(ECH-*stat-t*BGE) **1–3**

*p*(ECH-*stat-t*BGE)	*M*_*n*,theo_/Da^a^	ECH:*t*BGE^b^	*M_n_*/Da^c^	*Đ*^c^	yield/%
**1**	3100	22:78	6200	1.5	89
**2**	2700	60:40	4900	1.6	92
**3**	2500	80:20	3100	1.3	quant.

a *M*_*n*,theo_ = (*n*_ECH_/*n*_NBu4Br_)·*M*_ECH_ + (*n*_*t*BGE_/*n*_NBu4Br_)·*M*_*t*BGE_.

b NMR spectroscopy in CDCl_3_.

c SEC in THF, calibrated with pMMA standards.

**Figure 1:**
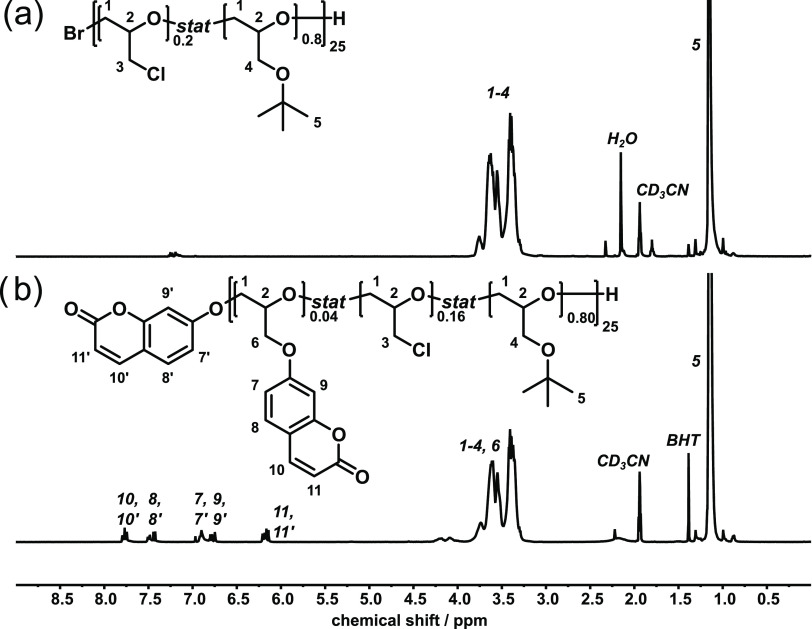
^1^H NMR spectra of (a) p(ECH-*stat-t*BGE) **1** and (b) of *p*(CumGE-*stat*-ECH-*stat*-*t*BGE) **4**. Measured in CD_3_CN.

The ^1^H NMR spectrum of **1** is shown in Figure 1a. The ECH/*t*BGE ratio was calculated for **1**–**3** from the ^1^H NMR signal intensity at 1.16 ppm for the *tert*-butyl group relative to the signals at 3.30 to 3.76 ppm and are in good agreement with the monomer ratio applied for the polymerization (Table 1, a detailed description of the monomer ratio calculation is shown in the Supporting Information).

Next, polymers **1**–**3** were used in Williamson ether synthesis using 7-hydroxycoumarin as a nucleophile and K_2_CO_3_ as a base in DMF. Depending on the ECH/*t*BGE ratio in the prepolymer, a conversion of 20–40% of ECH units could be achieved. A higher content of ECH in the polymer leads to increased conversions due to a lower amount of sterically demanding *tert*-butyl groups. The conversion of ECH to CumGE was enhanced further up to 54%, by increasing the equivalents of 7-hydroxycoumarin from 1.1 equiv to an excess of 2.2 equiv The ^1^H NMR spectrum of *p*(CumGE-*stat*-ECH-*stat*-*t*BGE) **4** is shown in Figure 1b. In addition to the *t*BGE and polymer backbone signals, new signals appear in the aromatic range between 6.14 and 7.79 ppm, which are assigned to the added coumarin groups. The substitution reaction also occurred at the bromine-terminated chain end (Scheme 1b). End-group and side-chain coumarin moieties can be distinguished in the NMR spectrum of polymer **4**. From this, the molecular weight of the polymer was calculated to be 3800 Da (a detailed description of the monomer ratio calculation and the determination of the molecular weight from NMR are shown in the Supporting Information). Due to broadening of the coumarin signals with increased degree of functionalization, end- and side-group coumarin could not be distinguished for polymers **5**–**8** and their molecular weights could only be obtained from SEC (Table 2).

**Table 2:** Analytical Data from NMR and SEC for the Coumarin Functionalization of *p*(ECH-*stat-t*BGE) **4**-**8**: Conversion and Resulting Monomer Ratio in Dependence of the Used Prepolymer and Equivalents of 7-Hydroxycoumarin (OH-Cum)

					*M*_*n*_/Da^b^		
*p*(CumGE-*stat*-ECH-*stat*-*t*BGE)	prepolymer	equiv OH-Cum	CumGE/*t*BGE/ECH^a^	conversion/%^a^	SEC^b^	NMR^a^	*Đ*^b^^a^
**4**	**1** (22:78)	1.1	4:78:18	18	5800	3800	1.8
**5**	**2** (61:39)	1.1	25:39:36	41	3100	n.a.	1.6
**6**	**2** (61:39)	2.2	31:38:31	50	3100	n.a.	1.5
**7**	**3** (80:20)	1.1	26:19:54	33	2900	n.a.	1.4
**8**	**3** (80:20)	2.2	43:20:37	54	3100	n.a.	1.4

a NMR spectroscopy in CD_3_CN.

b SEC in THF, calibrated with pMMA standards.

Uniform-sized microgels (MG **I–IV**) were prepared by photo-cross-linking of the *p*(CumGE-*stat*-ECH-*stat*-*t*BGE) prepolymers **4**–**7** in a miniemulsion. For this purpose, the respective prepolymer and benzophenone as photosensitizers were dissolved in toluene and emulsified in a solution of SDS in water by ultrasonication. Hexadecane was added in order to minimize broadening of the size distribution of the oily droplets by Ostwald ripening.^45^ The polymers were cross-linked by irradiation with a UV-LED module at λ = 365 nm, inducing the dimerization of coumarin in emulsion droplets and leading to the formation of microgels **I**–**IV**. Prepolymer **8**, with the highest coumarin content of 43%, could not be dissolved in toluene and could therefore not be used for microgel synthesis with the presented method.

**Figure 2:**
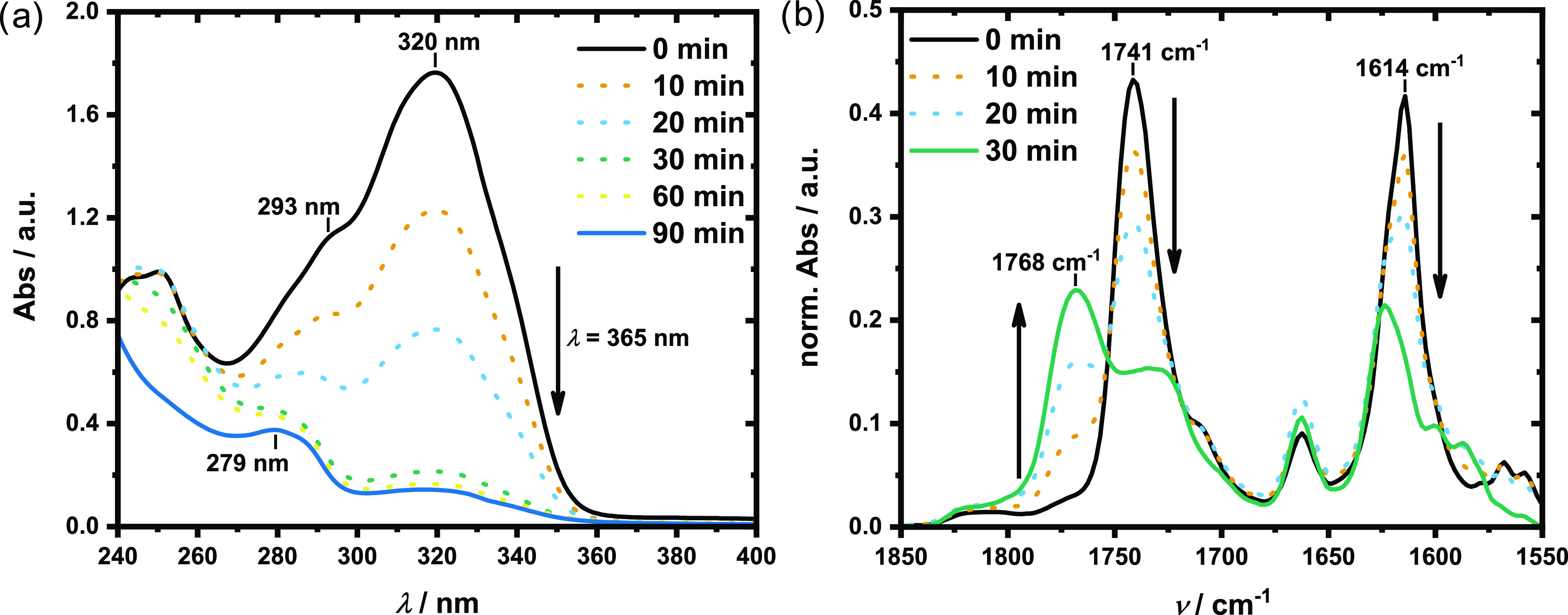
Photo-cross-linking kinetics of *p*(CumGE-*stat*-ECH-*stat*-*t*BGE) **4** upon irradiation at λ = 365 nm. (a) UV–vis spectra, measured in MeCN and taken between 0 and 90 min of irradiation, showing a decrease in the characteristic coumarin band at 320 nm, while the band at 279 nm increases in intensity. (b) FTIR spectra, recorded between 0 and 30 min of irradiation, show the increase of an additional C=O stretching band at 1741 cm^–1^, while the C=C stretching band at 1614 cm^–1^ decreases with increasing reaction time.

Photo-cross-linking kinetics of *p*(CumGE-*stat*-ECH-*stat*-*t*BGE) **4** were studied by UV–vis and IR spectroscopy (Figure 2). With increasing irradiation duration, the adsorption band at 320 nm in the UV–vis spectrum decreases while the band at 293 nm shifts to 279 nm, resulting from the [2π + 2π] cycloaddition of two coumarin groups. The addition of benzophenone as the photosensitizer facilitates the dimer formation (Figure S6). As seen in Figure 2a, an irradiation time of 30 min of polymer **4** with the addition of benzophenone is sufficient to provide the highest achievable yield of coumarin dimers. The success of the cross-linking reaction is further supported by IR spectroscopy. Besides the carbonyl stretching band at 1741 cm^–1^, a second band at 1768 cm^–1^ arises with increasing irradiation time, corresponding to the nonconjugated lactone rings in coumarin dimers (Figure 2b). Additionally, the C=C stretching band at 1614 cm^–1^ decreases with increasing cyclobutane content. Although a longer irradiation time than 30 min does not result in increased dimer formation, the cross-linking reaction is not quantitative, as indicated by the remaining peak of coumarin monomers at 1741 cm^–1^.

**Figure 3:**
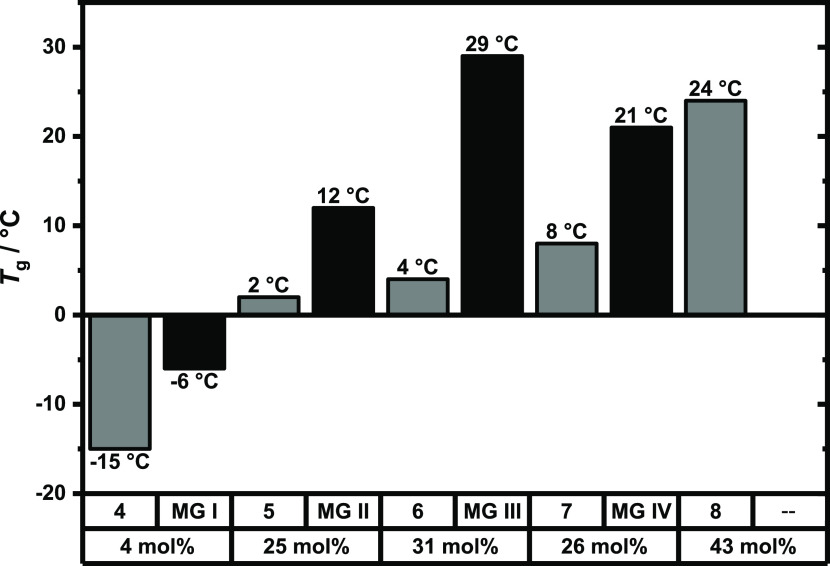
Comparison of glass transition temperatures of *p*(CumGE-*stat*-ECH-*stat*-*t*BGE) **4**–**8** (gray rectangle) with the respective microgel MG **I**–**IV** (black rectangle). From polymer **8**, no microgel was synthesized.

Microgels **I**–**IV** and the polymers **4**–**8** were analyzed by differential scanning calorimetry (DSC). In Figure 3, the glass transition temperatures *T*_g_ of microgels and their prepolymers are depicted. Cross-linking leads to an increased *T*_g_ in every case, and the glass transition temperature of **I**–**IV** ranges from −6 to 29 °C. Generally, *T*_g_ increases with higher coumarin functionalization due to increased π-stacking interactions. Therefore, the rigidity of the microgel can be predetermined by the coumarin content in the prepolymer.

**Figure 4:**
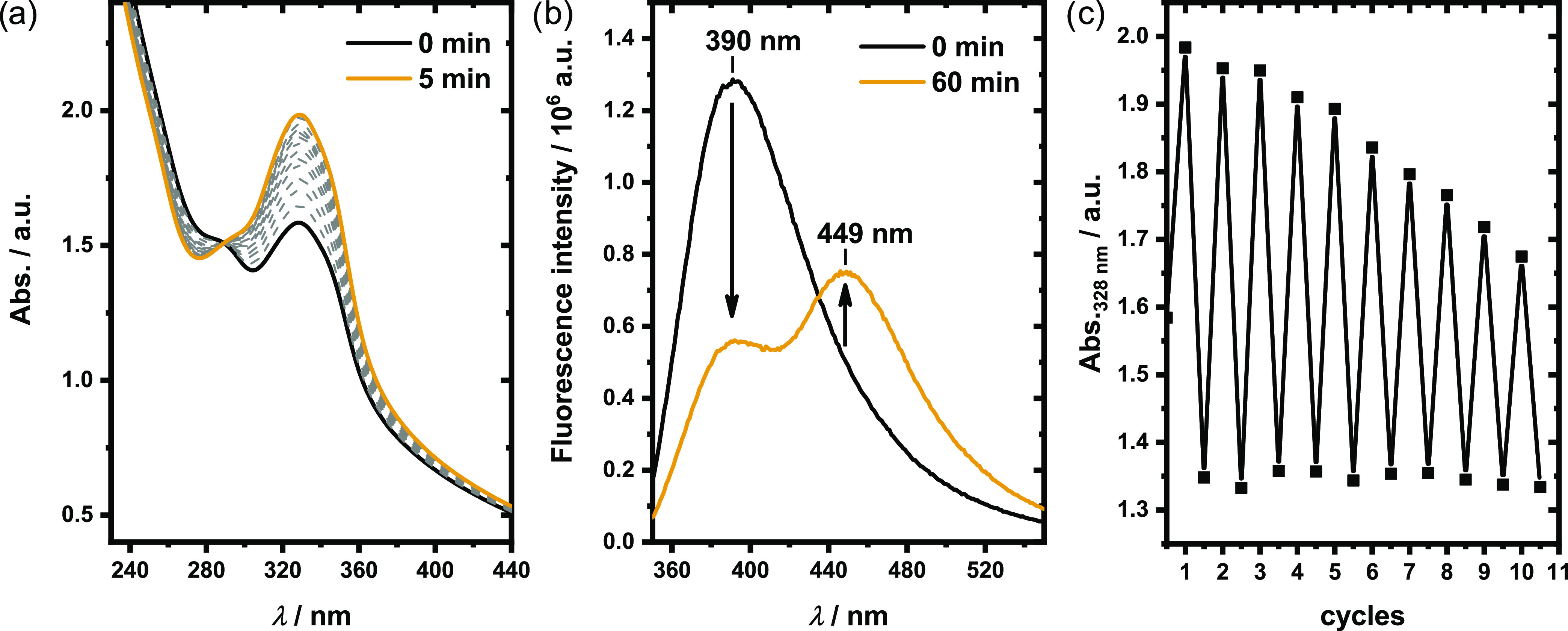
(a) UV–vis and (b) fluorescence emission spectra (λ_ex_ = 320 nm) of the photocleavage of coumarin cross-links in MG **II** by irradiation at 254 nm (in H_2_O); (c) reversibility of photo-cross-linking and -cleavage over 10 cycles analyzed by the absorption at 328 nm using UV–vis spectroscopy.

In order to reverse the photodimerization by irradiation at smaller wavelengths, we irradiated MG **II** at 254 nm under stirring. Photocleavage of MG **II** was monitored by UV–vis and fluorescence spectroscopy. The absorption spectrum in Figure 4a with its isosbestic point shows that irradiation at λ = 254 nm for 10 min is sufficient to reform coumarin’s conjugated π-electron system, as indicated by an increase of intensity at 320 nm. In the fluorescence spectrum in Figure 4b, photocleavage leads to a decrease of the band at 390 nm while a new band at 449 nm arises (λ_ex_ = 320 nm, see also confocal image in Figure S7). Irradiation-controlled cross-linking and cleavage of the cross-links could be repeated several times as demonstrated in Figure 4c. However, the absorption of the coumarin structure decreased to a value of only 50% of the initial intensity within 10 cycles, indicating some loss of reversibility by photobleaching.

**Scheme 2:**
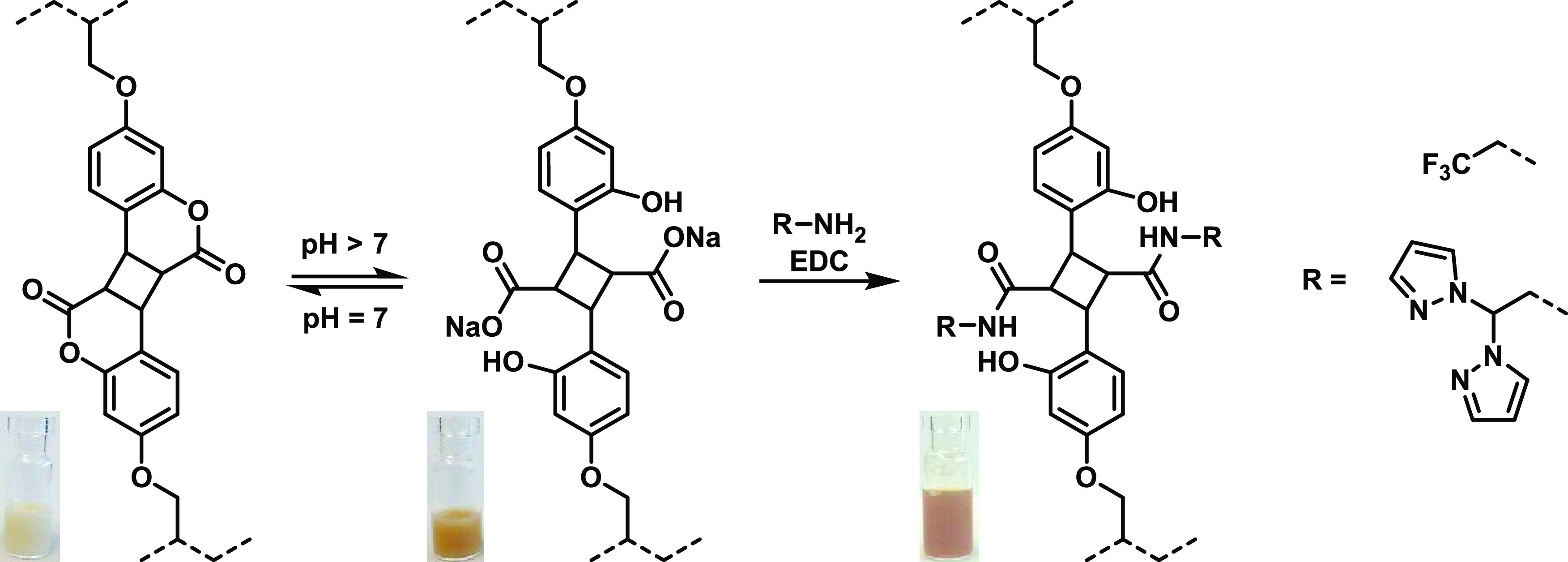
Reversible Lactone Hydrolysis of Coumarin Dimers in Microgels Increasing the pH using 2 M NaOH leads to the formation of sodium carboxylates while the color of the dispersion turns to red–brown. Upon neutralization, the lactone ring is reformed and the dispersion changes back to white. Microgels with open-ring coumarin cross-links were treated with two primary amines in the presence of EDC to form amides.

Coumarin dimers react rapidly with nucleophiles like amines and alcohols to give cyclobutane dicarbonyl derivatives.^36^ In the following, we demonstrate that this reaction opens an easy way for further functionalization of the microgels described above. First, we show the lactone ring opening under basic conditions and in a subsequent reaction the binding of trifluoroethylamine as a model substrate and 2,2-dipyrazol-1-yl-ethanamine (pz-NH_2_) as depicted in Scheme 2.

**Figure 5:**
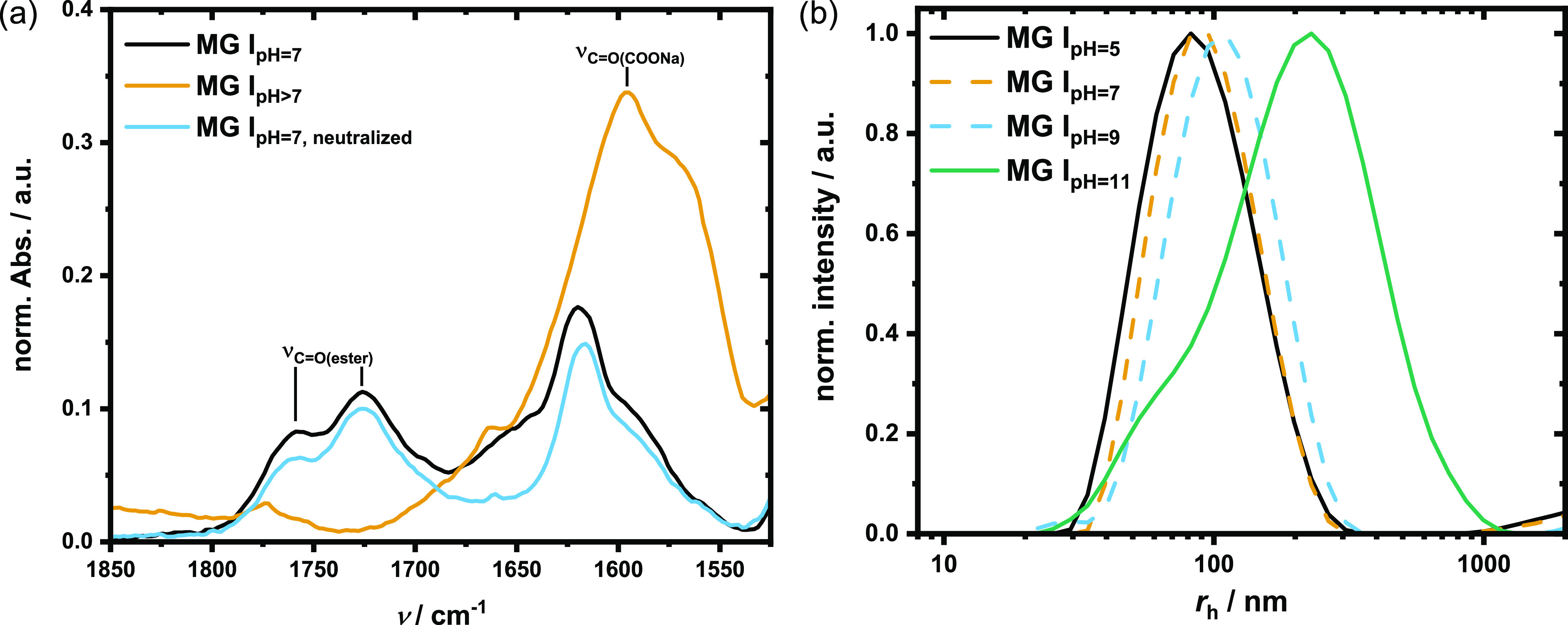
Base-induced, reversible lactone hydrolysis of coumarin dimers. (a) FTIR spectra of MG **I** in neutral and caustic media. Black: MG **I** at pH = 7, showing the lactone carbonyl band at 1759 cm^–1^; gold: increasing the pH with 2 M NaOH leads to lactone hydrolysis and the formation of a carboxylate band at 1595 cm^–1^; blue: reformation of lactone by neutralization of the dispersion. (b) Size distribution of MG **I** at pH = 5–11, as determined by light scattering.

When MG **I** and **II** were dispersed in caustic water, the formation of carboxylates could be observed by a change of color from white to red–brown as well as the rise of a carboxylate band at 1595 cm^–1^ and disappearance of the ester C=O stretching band at 1759 cm^–1^ in the FTIR spectrum (Figure 5a). Furthermore, hydrolysis resulted in swelling of the microgel, as shown in Figure 5b, by the shift of the size distribution toward larger hydrodynamic radii (*r*_h_). The lactone rings could be reformed by neutralizing the dispersion, proven by the reappearance of the ester band at 1759 cm^–1^ (Figures 5a and S8).

**Figure 6:**
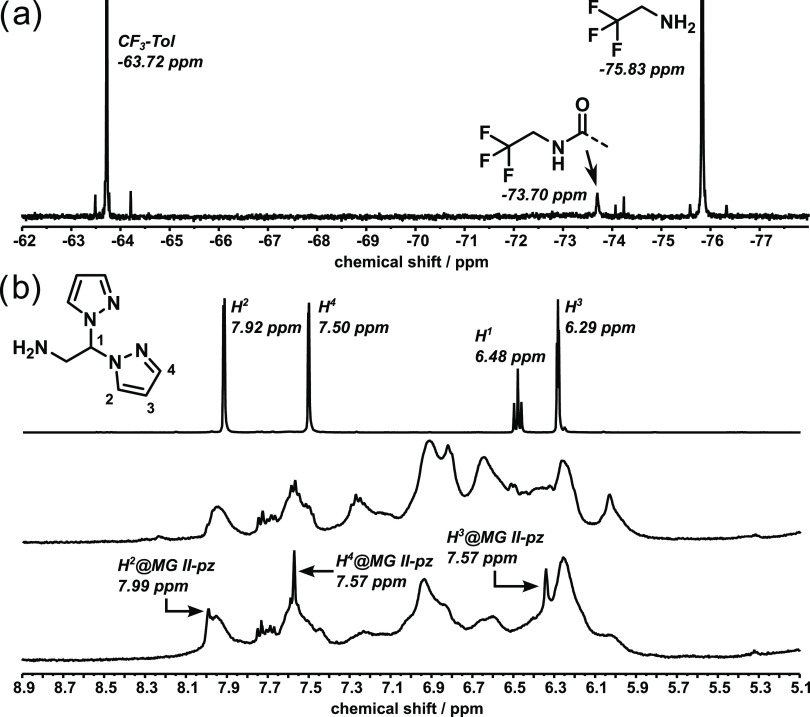
NMR spectra of the functionalization of MG **II**. (a) ^19^F NMR spectrum of a mixture of MG **II**-CF_3_, trifluoroethylamine and trifluorotoluene (CF_3_-Tol) as standard. (b) ^1^H NMR spectra of the starting materials 2,2-dipyrazol-1-yl-ethanamine and MG **II** as well as functionalized MG **II**-pz. All spectra were measured in DMSO-*d*_6_.

Amidation of MG **II** occurred in basic media with trifluoroethylamine (MG **II**-CF_3_) or pz-NH_2_ (MG **II**-pz) in the presence of EDC for 24 h at room temperature. After purification and isolation, the functionalized microgels were analyzed by NMR spectroscopy. Figure 6a shows a ^19^F NMR spectrum of **II**-CF_3_ with some added trifluoroethylamine, demonstrating the shift of the trifluoromethyl group from −75.83 ppm in the starting material to −73.70 ppm in **II**-CF_3_. In Figure 6b, the ^1^H NMR spectra of pz-NH_2_, **II**, and **II**-pz are shown. Although the signals of the pyrazolyl moiety overlap with the coumarin signals, three peaks at 6.34, 7.57, and 7.99 ppm are clearly visible in the spectrum of **II**-pz, which are not present before the functionalization. Moreover, the signals are slightly shifted, compared to the spectrum of the isolated amine. Thus, the functionalization reaction was successful.

**Figure 7:**
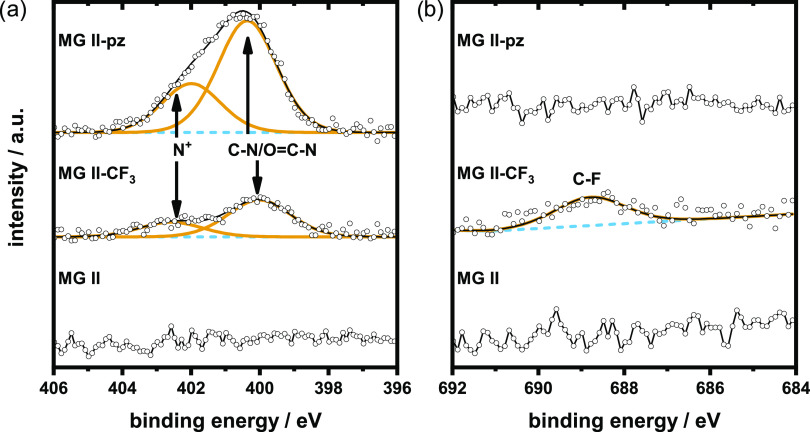
(a) N 1s and (b) F 1s XP spectra of microgels MG **II** (bottom), MG **II**-CF_3_ (center), and MG **II**-pz (top), respectively.

X-ray photoelectron spectroscopy (XPS) was used to further investigate the microgels. N 1s and F 1s XP spectra of MG **II**, MG **II**-CF_3_, and MG **II**-pz are shown in Figure 7. For both functionalized gels, a new peak at 400.0 eV corresponding to amides arises, which is not present in the spectrum of MG **II**. Thus, the covalent binding of both primary amines to hydrolyzed lactones was successful.^46^ Moreover, the peak at 402.3 eV corresponds to protonated nitrogen atoms.^47^ The intensity of the amide peak in MG **II**-pz is increased compared to MG **II**-CF_3_ due to an overlap with the signal of pyrazol groups at 400.4 eV.^48^ Only for MG **II**-CF_3_, a peak at 688.8 eV in the F 1s spectrum is visible, corresponding to the trifluoromethyl moiety.^49^ Signals at 285.0, 286.6, and 288.7 eV, assigned to C–H, CO/CN and O=C–O/O=C–N groups, in the C 1s XP spectrum (Figure S11) further support the postulated microgel composition. Lastly, Cl 2p_3/2_ peaks at 200.4 eV show the presence of residual ECH groups. Weak signals at 197.9 eV are assigned to Cl^–^ ions. These can be assigned to the XPS-induced degradation of organic halides.^50^ However, this peak is most pronounced in the spectrum of MG **II**-CF_3_, indicating some formation of ammonium chloride groups stemming from the addition of trifluoroethylamine to residual ECH.

**Figure 8:**
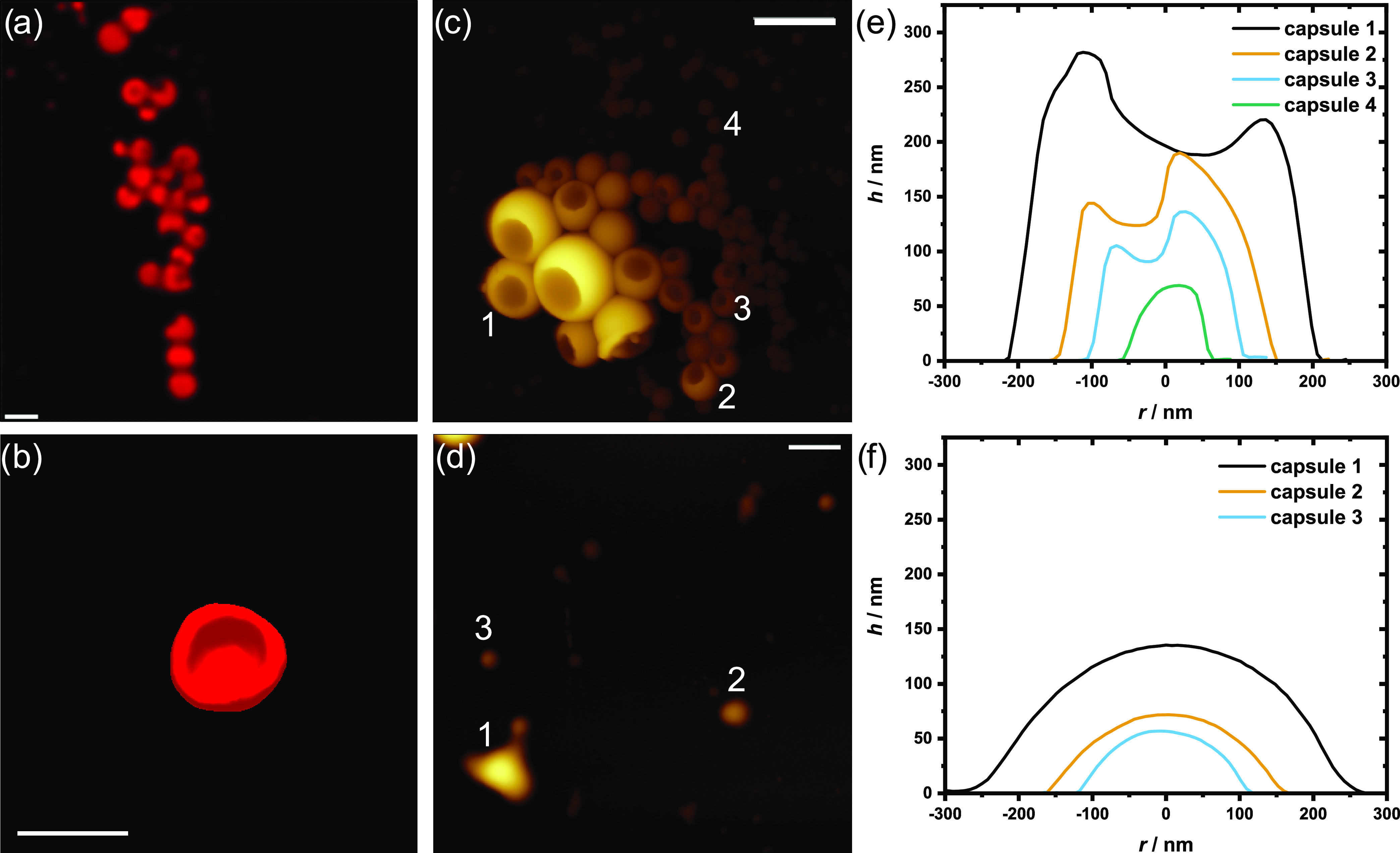
Microscopic images of MG **II**. (a) Confocal and (b) surface-rendered STED microscopy images of Nile red-stained MG **II** (scale bar 1 μm). (c) AFM image of MG **II** before and (d) after photocleavage of the cross-links by irradiation at 254 nm (scale bar 0.5 μm). (e) Height profiles of selected MG **II** capsules before and (f) after photocleavage. The generated gel capsules have a constant aspect ratio between the cavity and the highest point of the gel in AFM measurements, regardless of capsule size.

So far, we described the synthetic scope of the coumarin functionalized prepolymers, whereas we prepared small gel objects of rather uniform finite size within the small droplets of a miniemulsion. However, we did not consider whether the gel particles templated the oil droplets simply with a uniform internal structure. Actually, regarding the high degrees of cross-linking expected for the high fraction of coumarin substituents ranging from 4 to 40% of the monomer units and regarding the fact that the polyglycidols demonstrate a certain amphiphilicity, such a simple spherical microgel structure would be surprising. Hence, we analyzed the microgels in the dry state by confocal, stimulated emission depletion (STED), and atomic force (AFM) microscopies. Although the least cross-linked MG **I** turned out to be too soft for microscopic analysis, dried MG **II** could be analyzed, revealing a deflated capsule structure (Figure 8). The aspect ratio between the highest point of the microgel and the lowest point of the cavity was obtained using height profiles from AFM images. Regardless of capsule size, the aspect ratio remains constant at 1.4. After irradiation at 254 nm, the cross-linking density of the microgels was reduced due to the cleavage of coumarin dimers and spherical microgels were obtained. We identify these structures in Figure 8b,c as that of collapsed microcapsules, which resemble stomatocytes.^51,52^ Its formation can be explained as the prepolymer concentrates at the surface of the oil droplets like a surfactant, where it gets cross-linked by the photodimerization. Upon drying, the capsule must collapse as the oil phase mostly consisting of toluene evaporates. It is also possible that the light used for cross-linking is attenuated and can therefore not fully penetrate the droplet to ensure even cross-linking of the preopolymers. The cross-linking process was carried out at λ = 365 ± 5 nm, as specified by the manufacturer of a UV lamp. We measured UV–vis absorption spectra of 7-methoxycoumarin, serving as a model compound representing polymerized CumGE, and determined the extinction coefficients ε at λ = 318 and 350 nm (Figure S12). Following Beer–Lambert’s law, the corresponding transmittances *T* were calculated to be 73.6 and 97.9%, respectively, at a penetration depth of 200 nm, which corresponds to the radius of the largest capsule of **II** analyzed via AFM (Figure 8e). Thus, no meaningful attenuation of light occurs and its intensity is sufficient for cross-linking across the entire volume of a droplet. Moreover, coumarin dimers formed during the cross-linking of **4** neither show absorbance above λ = 360 nm, as seen in Figure 2a. The generated gel capsules have a constant aspect ratio between the cavity and the highest point of the gel in AFM measurements, regardless of capsule size (Figure 8e). If significant light attenuation took place in a droplet, the wall thicknesses would remain constant as the penetration depth of light would remain the same, leading to a capsule size-dependent aspect ratio. Therefore, we conclude that the formation of capsules, rather than homogeneously cross-linked microgels, is a result of the orientation of prepolymers at the water/oil interface. Photocleavage of cross-links destroys the structure, and as the long cleavage is not perfect, an amorphous object remains.

The unraveling of the capsule morphology also allows for further discussion of the functionalization of MG **II**. The reactivities of both primary amines used are assumed to be similar and both nucleophiles form amides with hydrolyzed lactones of coumarin dimers, as seen in the presence of amide peaks in the N 1s XP spectra (Figure 7a). Coumarin dimers are mostly present at the exterior of synthesized capsules through templating of the oil–water interface during cross-linking, while hydrophobic ECH and *t*BGE are mostly located in the interior. Assuming an average bond length of 1.3 Å for C–F and pyrazolyl bonds, trifluoromethyl has an approximate diameter of 2.0 Å, while the diameter of the bispyrazolyl group is estimated to be at least 5 Å. Trifluoroethylamine can penetrate the capsule shells after lactone hydrolysis due to its lower size and also attack residual ECH. In this reaction, ammonium chloride moieties are formed, which are visible as Cl^–^ ions in the Cl 2p XP spectra of MG **II**-CF_3_ (Figure S11b). On the contrary, pz-NH_2_ is too large to enter the capsule and can only react with hydrolyzed lactones, which is why the chloride ion peak is much less pronounced and can be attributed to the aforementioned degradation of organic halides.

## Conclusions

Not originally intended, we discovered the formation of soft nanocapsules by photo-cross-linking of coumarin-functionalized poly(*tert*-butyl glycidyl ether) by the observation of stomatocyte-like structures, when the objects formed by photo-cross-linking were imaged in the dry state after the originally enclosed solvent evaporated. Such structures are highly characteristic for dried nanocapsules, e.g., formed from silica,^53−55^ from blockcopolymer polymersomes,^56−58^ and their formation has been discussed intensively for the osmotic collapse of microcapsules.^59^ We explain the formation of a tight capsule wall at least partly by a distinct amphiphilic character of the prepolymers with their polar polyether backbone. However, the particularly new aspect of the capsules described here, which needs further studies, is the fact that their walls demonstrate (i) flexibility combined with high mechanical stability as they survive drying, (ii) reversible photoresponsiveness as they can be disintegrated and rebuilt, and furthermore, they can be (iii) modified for hydrophilicity and their permeability by polar molecules. Modification is offered by hydrolysis of the lactone groups and functionalization with nucleophiles. Yet, the coumarin dimers do not only serve as light switchable cross-links; in addition, functional molecules, such as peptides, can be tethered to them. At the same time, the cross-links get stabilized. The polyglycidol itself is a nontoxic, biocompatible building block approved by the Food and Drug Administration (FDA).^60,61^ In summary, we propose the coumarin-functionalized polyglycidyl ethers as versatile building blocks for future nanocarrier systems.

## Experimental Section

### Materials

*tert*-Butyl glycidyl ether (*t*BGE, 99%, Sigma-Aldrich) and epichlorohydrin (ECH, 99%, Sigma-Aldrich) were dried over CaH_2_ (93%, Thermofisher) for 24 h under a N_2_ atmosphere and distilled before use. 2,2-Dipyrazol-1-yl-ethanamine (pz-NH_2_) was synthesized following the procedure of Reger et al.^62^ All other chemicals were obtained from commercial sources and were used without further purification.

#### Synthesis of *p*(ECH-*stat-t*BGE) **1**

NBu_4_Br (0.75 g, 2.31 mmol, 0.04 equiv) was dried under vacuum for 3 h and then dispersed in anhydrous toluene (30 mL) under a N_2_ atmosphere. ECH (1.13 g, 12.19 mmol, 0.2 equiv) and *t*BGE (6.02 g, 46.26 mmol, 0.8 equiv) were added. The mixture was cooled to 0 °C using an ice bath, and then Al(*i*Bu)_3_ (1.1 M in toluene, 6.2 mL, 6.91 mmol, 0.12 equiv) was rapidly added. After 15 min, the ice bath was removed and the polymerization was continued for 2 h at room temperature. The reaction was quenched by the addition of MeOH (2 mL). Solvents were removed under reduced pressure, and the crude polymer was redissolved in DCM and washed 3 times with 2 M NaOH solution. The organic phase was dried over MgSO_4_. DCM was removed under reduced pressure and *p*(ECH-*stat-t*BGE) **1** was obtained as a colorless oil (6.36 g, 89%, 22 mol % ECH, 78 mol % *t*BGE). ^1^H NMR (CD_3_CN): δ = 3.76–3.30 (m, 10H^1–4^), 1.16 (s, 9H^5^) ppm. ^13^C NMR (CD_3_CN): δ = 80.2, 73.4, 71.1, 70.9, 62.6, 62.3, 45.4, 27.9 ppm. *M*_*n*,SEC_ = 6200 Da, *Đ* = 1.5. *T*_g_ = −36 °C. Polymers **2** and **3** were synthesized accordingly, and experimental and analytical details are to be found in the Supporting Information.

#### Synthesis of *p*(CumGE-*stat*-ECH-*stat*-*t*BGE) **4**

p(ECH-*stat-t*BGE) **1** (3.00 g, 4.89 mmol ECH, 1.0 equiv ECH) was dissolved in anhydrous DMF (20 mL) in a three-neck flask, equipped with a reflux condenser. K_2_CO_3_ (2.03 g, 14.67 mmol, 3.0 equiv) and 7-hydroxycoumarin (0.87 g, 5.38 mmol, 1.1 equiv) were added to the polymer solution. The mixture was heated to 65 °C for 80 h and then allowed to cool to room temperature. The precipitate was removed by filtration, and the polymer solution was diluted with 50 mL of water and extracted with DCM 3 times. The combined organic phases were dried over MgSO_4_. DCM was removed under reduced pressure and *p*(CumGE-*stat*-ECH-*stat*-*t*BGE) **4** was obtained as a yellow–green oil (3.13 g, 4 mol % CumGE, 18 mol % ECH, 78 mol % *t*BGE). ^1^H NMR (CD_3_CN): δ = 7.77 (m, 2H^10,10′^), 7.50 (d, 1H^8^), 7.43 (d, 1H^8′^), 6.90 (m, 2H^7,9^), 6.80–6.74 (m, 2H^7′,9′^), 6.20 (d, 1H^11^), 6.15 (d, 1H^11′^), 4.18–3.36 (m, 15H^1–4,6^), 1.15 (s, 9H^5^) ppm. ^13^C NMR (CD_3_CN): δ = 144.9, 130.5, 130.2, 113.8. 113.2, 103.5, 80.3, 73.4, 71.1, 70.9, 62.6, 62.3, 45.4, 27.9 ppm. *M*_*n*,NMR_ = 3800 Da. *M*_*n*,SEC_ = 5800 Da, *Đ* = 1.8. *T*_g_ = −15 °C. Polymers **5**–**8** were synthesized accordingly, and experimental and analytical details are to be found in the Supporting Information.

### Preparation of MG **I**–**IV**

*p*(CumGE-*stat*-ECH-*stat*-*t*BGE) **4**–**7** (0.25 g), hexadecane (0.02 g), and benzophenone (0.009 g) were dissolved in toluene (0.88 g). SDS (0.002 g) was dissolved in water (4.0 g), and the solution was passed through a syringe filter and added to the polymer solution. A miniemulsion was prepared using a Branson Ultrasonifier 450 for 15 min (output control 3, duty cycle 50%). The emulsion was then irradiated with a UV-LED cube (λ = 365 nm) for 30–90 min while vigorously stirring with a mechanical stirrer. After the reaction, the microgel was purified by at least five cycles of dialysis (MWCO = 10,000 Da) in water (2.5–5.0 L per cycle) followed by lyophilization.

#### Synthesis of MG **II**-CF_3_

MG **II** (0.050 g, 0.10 mmol CumGE, 1.0 equiv CumGE) was dispersed in water (2 mL), and then a few droplets of 2 M NaOH solution, 2,2,2-trifluoroethylamine (9.8 μL, 0.012 g, 0.13 mmol, 1.3 equiv), and EDC (0.042 g, 0.22 mmol, 2.2 equiv) were added. The mixture was stirred for 24 h at room temperature. Functionalized microgel MG **II**-CF_3_ was obtained after five cycles of dialysis (MWCO = 10,000 Da) in water (2.5–5.0 L per cycle) followed by lyophilization. The ^19^F NMR spectrum of the product can be found in Figure 6a.

#### Synthesis of MG **II**-pz

MG **II** (0.170 g, 0.36 mmol CumGE, 1.0 equiv CumGE) was dispersed in water (5 mL), and then a few droplets of 2 M NaOH solution, 2,2-dipyrazol-1-yl-ethanamine (0.095 g, 0.54 mmol, 1.5 equiv), and EDC (0.145 g, 0.76 mmol, 2.1 equiv) were added. The mixture was stirred for 24 h at room temperature. Functionalized microgel MG **II**-pz was obtained after five cycles of dialysis (MWCO = 10,000 Da) in water (2.5–5.0 L per cycle) followed by lyophilization. The ^1^H NMR spectrum of the product can be found in the Supporting Information (Figure S5).

## Supporting Information Available

Instruments; methods; additional synthetic procedures; further analyses of polymers and microgels; calculation of monomer ratios and calculation of molecular weight by NMR (PDF)

## Notes

A preliminary version of this work was submitted as an unformatted preprint to the ChemRxiv (Cambridge Open Engage).^63^

## Abbreviations

AFM: atomic force microscopy
Al(*i*Bu)_3_: triisobutylaluminum
CD_3_CN: acetonitrile (deuterated)
BHT: 2,6-di-*tert*-butyl-4-methylphenol
CDCl_3_: chloroform (deuterated)
CumGE: coumarin glycidyl ether
DCM: dichloromethane
DLS: dynamic light scattering
DMF: dimethylformamide
DMPA: 2,2-dimethoxy-2-phenylacetophenone
DMSO-*d*_6_: dimethyl sulfoxide (deuterated)
DSC: differential scanning calorimetry
ECH: epichlorohydrin
EDC: 1-ethyl-3-(3-dimethylamino-propyl)carbodiimide
IR: infrared
K_2_CO_3_: potassium carbonate
LED: light emitting diode
MeCN: acetonitrile
MeOH: methanol
MMA: methyl methacrylate
MWCO: molecular weight cutoff
N(Bu)_4_Br: tetra-butylammonium bromide
NaOH: sodium hydroxide
OH-Cum: 7-hydroxycoumarin
pz-NH_2_: 2,2-dipyrazol-1-yl-ethanamine
rt: room temperature
SEC: size exclusion chromatography
STED: stimulated emission depletion
*t*BGE: *tert*-butyl glycidyl ether
THF: tetrahydrofuran
UV: ultraviolet
