# Supplementary material for: Photo-cross-linked and pH-Switchable Soft Polymer Nanocapsules from Polyglycidyl Ethers
Source: Macromolecules. 2023 Dec 29;57(2):707–18. doi: 10.1021/acs.macromol.3c01698 (PMC10810002; doi:10.1021/acs.macromol.3c01698)
Supplement: Supplementary file 1 — ma3c01698_si_001.pdf [file ma3c01698_si_001.pdf]

Photocrosslinked and pH-switchable Soft Polymer Nanocapsules from Polyglycidyl Ethers

Stefan Engel[1,2,‡], Pascal M. Jeschenko[2,3,†,‡,], Marcel van Dongen[2], Jonas C. Rose[2], Dominic Schäfer[4], Michael Bruns[5], Sonja Herres-Pawlis[4], Helmut Keul[2] and Martin Möller[1,2,3,*]*

[1] Institute of Technical and Macromolecular Chemistry (ITMC), RWTH Aachen University Worringerweg 2, D-52074, Aachen, Germany

[2] DWI - Leibniz-Institute for Interactive Materials, Forckenbeckstraße 50, D-52074, Aachen, Germany

[3] Max Planck School Matter to Life, Jahnstraße 29, D-69120, Heidelberg, Germany

[4] Institute of Inorganic Chemistry (IAC), RWTH Aachen University, Landoltweg 1, D-52074, Aachen, Germany

[5] Institute for Applied Materials and Karlsruhe Nano Micro Facility, Karlsruhe Institute of Technology, Hermann-von-Helmholtz-Platz 1, D-76344, Eggenstein-Leopoldshafen, Germany

[†] Max Planck Institute for Medical Research, Jahnstraße 29, D-69120, Heidelberg, Germany *(current address)*

Table of Contents

Instruments and methods 2

Synthetic Procedures and Analytics 6

Calculation of Monomer Ratios in p(ECH-*stat*-*t*BGE) 1-3 and p(CumGE-*stat*-ECH-*stat*-*t*BGE) 4-8 10

Calculation of the Molecular Weight of p(CumGE-*stat*-ECH-*stat*-*t*BGE) 4 by NMR 11

Optimization of Microgel Synthesis 13

IR Spectra of MG I at Different pH 15

Differential Scanning Calorimetry 16

X-ray Photoelectron Spectroscopy 18

Calculation of Light Penetration Depth During Photocrosslinking 19

References 21

Instruments and methods

NMR spectroscopy. ^{1}H-, ^{19}F- and ^{13}C-NMR spectra were recorded on a Bruker Avance III 400 spectrometer (400, 400 and 100 MHz, respectively; Bruker Corporation, Billerica, MA, USA) and are reported as follows: chemical shift δ (ppm) (multiplicity, number of protons, assignment). Deuterated Acetonitrile (CD_3CN, $\delta_H = 1.94$ ppm, $\delta_C = 118.3$ ppm) and dimethylsulfoxide (DMSO-d_6, $\delta_H = 2.50$ ppm, $\delta_C = 39.52$ ppm) were used as an internal standard for ^{1}H- and ^{13}C-NMR and trifluorotoluene ($\delta_F = -63.72$ ppm) for ^{19}F-NMR. Chemical shifts are reported in ppm to the nearest 0.01 ppm for ^{1}H- and ^{19}F-NMR and the nearest 0.1 ppm for ^{13}C-NMR.

Size-exclusion chromatography. Molecular weights (M_n and M_w) and molecular weight distributions (*Đ*) were determined by SEC. Analyses were carried out with tetrahydrofuran (THF, HPLC grade, Carl Roth, Karlsruhe, Germany) as eluent using an HPLC pump (PU-2080plus, Jasco, Gross-Umstadt, Germany) equipped with a refractive index detector (RI-2031plus, Jasco, Gross-Umstadt, Germany). The sample solvent contained 250 mg·mL^{-1} 3,5-di-*tert*-4-butylhydroxytoluene (BHT, ≥99%, Sigma Aldrich Chemie GmbH, Steinheim, Germany) as internal standard. One pre-column (8 mm × 50 mm) and four SDplus gel columns (8 mm × 300 mm, SDplus, MZ Analysentechnik, Mainz, Germany) were used at a flow rate of 1.0 mL·min^{-1} at 20 °C. The diameter of the gel particles measured 5 µm, the nominal pore widths were 50, 10^2, 10^3 and 10^4 Å. Calibration was performed using narrowly distributed poly(methyl methacrylate) standards (PSS Polymer Standards Service GmbH, Mainz, Germany) from 600 to 576,000 Da. The results were evaluated using the PSS WinGPC UniChrom software (Version 8.1.1).

UV-Vis irradiation. Crosslinking of prepolymers **4-7** was performed using a UV-LED module (λ = 365 nm, irradiance: 1000 mW/cm², power input 50 W, Serie L XS, Opsytec Dr. Gröbel, Ettlingen, Germany). Two UV handlamps (λ = 254 nm, irradiance: 500 µW/cm², 2·6 W power input, Opsytec Dr. Gröbel, Ettlingen, Germany) were used for photocleavage. All reaction mixtures used in irradiation experiments were stirred using a magnetic stirring bar.

UV-Vis spectroscopy. UV-Vis spectra were recorded on a JASCO V-630 spectrophotometer connected to a Huber Pilote-one 'petite fleur' thermostate using silicone oil (temperature range -40 °C to 110 °C) as thermofluid. Absorbance (Abs. in arb. units) was measured in quartz glass cuvettes (d = 10 mm) with a scan speed of 1000 nm·min^{-1} at fast response (data pitch = 1 nm) at T = 25 °C. Spectra analyses were evaluated using the JASCO spectra

manager software (Version 2). Samples of prepolymers were analyzed in MeCN and microgel samples dispersed in H_2O or in MeCN.

Infrared spectroscopy. IR spectra were carried out on a ThermoNicolet FT-IR Nexus spectrometer and are recorded on a silicon crystal (ThermoNicolet, Smart SplitPEA). Transmission maxima are reported in wavenumbers (cm^{-1}), only selected intensities are reported.

Fluorescence spectroscopy. Emission spectra were carried out in four-window quartz glass cuvettes on a spectrofluorometer FluoroMax-4 (Horiba Jobin Yvon GmbH, Longjumeau, France). The emission was measured from 340 – 700 nm using an excitation wavelength of $\lambda = 320$ nm.

Differential scanning calorimetry. The glass transition temperature T_g of prepolymers **1-8** and dried microgels **I-IV** were analyzed by differential scanning calorimetry (DSC). Samples of 5 – 10 mg (weighed on a Sartorius Balance CP2P; accuracy ±1 µg) in aluminum crucibles with lid were measured on a Perkin Elmer DSC 8500 equipped with a controlled liquid nitrogen cooling accessory (CLN2, setpoint: -80 °C / -110 °C). The measurements were performed under nitrogen between -80 °C and 60 °C for **1-3** and between -50 °C and 150 °C for **4-8** and **I-IV** with a heating rate of 10 $K \cdot min^{-1}$ for the first and 20 $K \cdot min^{-1}$ for the second heating cycle.

Atomic force microscopy. Samples for AFM measurements were prepared on a silicon wafer. First, the wafer was hydrophilized in a PVA Tepla Plasma System 100 (O_2, 5 min, 200 W). Microgel with a concentration of 1 mg/mL was spin coated on the wafer using a Laurell WS-650SZ-6NPP/LITE (1200 rpm, 1 min). AFM images were measured using tapping mode (Agilent 5500 SPM/AFM, Agilent Technologies) under ambient conditions. The standard silicon cantilevers used had a spring constant of 5 – 37 N/m and an oscillation frequency of 96 – 175 kHz. Data were processed using Gwyddion software, version 2.44.

Nile red staining. For confocal microscopy MG **II** was stained with Nile red. Therefore, 0.5 mL of a 1 mg·mL^{-1} aqueous dispersion of MG **II** was stirred with 5 µL of a 0.3 M solution of Nile red in acetone for 30 minutes at room temperature. To remove excessive Nile red, the stained microgel was dialyzed against water (MWCO: 500 – 1000 Da).

Confocal and super-resolution microscopy imaging. Confocal and stimulated emission depletion (STED) microscopies were performed with a Leica SP8 Tandem system, equipped with a 93x glycerin objective. For confocal imaging of microgels, samples were mounted onto cover slips (#1.5, Paul Marienfeld GmbH & Co. KG) and covered with a UV-transmissible 1 mm thick quartz microscope slide (Electron Microscopy Sciences) sealed with oil to prevent evaporation. Afterward, samples were excited with a photodiode 405 (λ = 405 nm, unlabeled) or white light laser (λ = 560 nm, Nile red) and signals were detected with a pinhole of 1 a.u. and a gating of 0.8 – 6 ns via a HyD detector (λ = 630 – 750 nm, unlabeled; λ = 570 – 700 nm, Nile red). For STED microscopy the zoom and resolution were adjusted to acquire a pixel size of approximately 20 nm and the signal was depleted by a pulsed laser with a wavelength of 775 nm. Additional z-resolution was gained by reducing the pinhole to 0.6 a.u. and a phase mask of approximately 30%. Images were subsequently deconvolved with the Huygens Professional software (Scientific Volume Imaging B.V.).

Dynamic light scattering. Size distributions of MG **I** at different pH were measured by DLS on a Zetasizer NanoZS (Malvern, UK) at a fixed angle of 173°. Very diluted samples were prepared with HCl and NaOH to adjust the respective pH 5, 7, 9 and 11. Samples were filtered using a 1.2 µm PET filter before measurement. The measurements were performed at 20 °C.

X-ray photoelectron spectroscopy. XPS measurements were performed using a K-Alpha$^+$ XPS spectrometer (Thermo Fisher Scientific, East Grinstead, UK). Data acquisition and processing using the Thermo Avantage software is described elsewhere.[1] All samples were

analyzed using a microfocused, monochromated Al Kα X-ray source (30 – 400 µm spot size). The spectra were fitted with one or more Voigt profiles. For intense peaks and/or peaks clearly evidenced by the peak shape, the binding energy uncertainty was around ±0.1 eV. In case of weak peaks and no direct justification by the peak shape, the uncertainty was set to ±0.2 eV. The analyzer transmission function, Scofield sensitivity factors, and effective attenuation lengths (EALs) for photoelectrons were applied for quantification.[2] EALs were calculated using the standard TPP-2M formalism.[3] All spectra were referenced to the C 1s peak of hydrocarbon at 285.0 eV binding energy controlled by means of the well-known photoelectron peaks of metallic Cu, Ag, and Au.

Synthetic Procedures and Analytics

Synthesis of p(ECH-*stat*-*t*BGE) 2. The experimental procedure is identical to that of p(ECH-*stat*-*t*BGE) **1** described in the main text. Polymer **2** was synthesized using $N(Bu)_4Br$ (0.70 g, 2.16 mmol, 0.04 eq.), Al(*i*Bu)$_3$ (1.1 M in toluene, 5.9 mL, 6.48 mmol, 0.12 eq.), ECH (3.00 g, 32.4 mmol, 0.6 eq.) and *t*BGE (2.81 g, 21.6 mmol, 0.4 eq.). Yield: 5.35 g (92%). 60 mol% ECH, 40 mol% *t*BGE. ^{1}H-NMR (CD_3CN): δ = 3.76 - 3.39 (m, 10H$^{1-4}$), 1.16 (s, 9H^5) ppm. ^{13}C-NMR (CD_3CN): δ = 79.8, 73.6, 71.1, 70.1, 62.6, 62.4, 45.0, 27.8 ppm. $M_{n,SEC}$ = 4,900 Da, $Đ$ = 1.6. T_g = -29 °C.

Synthesis of p(ECH-*stat*-*t*BGE) 3. The experimental procedure is identical to that of p(ECH-*stat*-*t*BGE) **1** described in the main text. Polymer **3** was synthesized using $N(Bu)_4Br$ (0.52 g, 1.62 mmol, 0.04 eq.), Al(*i*Bu)$_3$ (1.1 M in toluene, 4.4 mL, 4.86 mmol, 0.12 eq.), ECH (3.00 g, 32.4 mmol, 0.8 eq.) and *t*BGE (1.05 g, 8.1 mmol, 0.2 eq.). Yield: 4.05 g (quant.). 80 mol% ECH, 20 mol% *t*BGE. ^{1}H-NMR (CD_3CN): δ = 3.76-3.39 (m, 10H$^{1-4}$), 1.16 (s, 9H^5) ppm. ^{13}C-NMR (CD_3CN): δ = 79.7, 73.6, 71.0, 70.0, 62.3, 45.0, 27.8 ppm. $M_{n,SEC}$ = 3,100 Da, $Đ$ = 1.3. T_g = 33 °C.

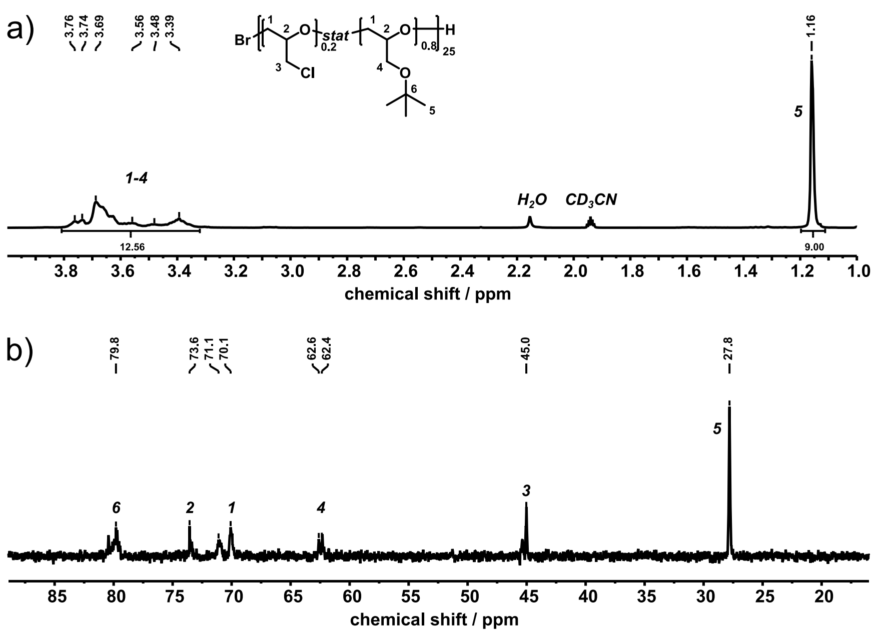

Figure S1. a) ^{1}H- and b) ^{13}C-NMR spectra of p(ECH-*stat*-*t*BGE) **2**, measured in CD_3CN.

Synthesis of p(CumGE-*stat*-ECH-*stat*-*t*BGE) 5. The experimental procedure is identical to that of p(CumGE-*stat*-ECH-*stat*-*t*BGE) **4** described in the main text. Polymer **5** was synthesized using p(ECH-*stat*-*t*BGE) **2** (3.00 g, 9.83 mmol ECH, 1.0 eq. ECH), K_2CO_3 (4.08 g, 29.94 mmol, 3.0 eq.) and 7-hydroxycoumarin (1.75 g, 10.81 mmol, 1.1 eq.). Yield: 3.3 g. 25 mol% CumGE, 36 mol% ECH, 39 mol% *t*BGE. ^{1}H-NMR (CD_3CN): δ = 7.70 (m, $2H^{10,10'}$), 7.41 (d, $2H^{8,8'}$), 6.79 (m, $4H^{7,7',9,9'}$), 6.13 (d, $2H^{11,11'}$), 4.05 - 3.36 (m, $15H^{1\text{-}4,6}$), 1.14 (s, $9H^{5}$) ppm. ^{13}C-NMR (CD_3CN): δ = 162.9, 161.5, 156.6, 144.6, 130.2, 113.7, 102.3, 79.9, 73.4, 70.8, 70.0, 62.3, 62.2, 45.0, 27.8 ppm. $M_{n,SEC}$ = 3,100 Da, $Đ$ = 1.6. T_g = 2 °C.

Synthesis of p(CumGE-*stat*-ECH-*stat*-*t*BGE) 6. The experimental procedure is identical to that of p(CumGE-*stat*-ECH-*stat*-*t*BGE) **4** described in the main text. Polymer **6** was synthesized using p(ECH-*stat*-*t*BGE) **2** (2.14 g, 7.01 mmol ECH, 1.0 eq. ECH), K_2CO_3 (2.91 g, 21.03 mmol, 3.0 eq.) and 7-hydroxycoumarin (2.5 g, 15.43 mmol, 2.2 eq.). Yield: 2.47 g. 31 mol% CumGE, 31 mol% ECH, 38 mol% *t*BGE. ^{1}H-NMR (CD_3CN): δ = 7.74 (m, $2H^{10,10'}$), 7.42 (d, $2H^{8,8'}$), 6.80 (m, $4H^{7,7',9,9'}$), 6.14 (d, $2H^{11,11'}$), 4.06 - 3.35 (m, $15H^{1\text{-}4,6}$), 1.13

(s, 9H^5) ppm. ^{13}C-NMR (CD_3CN): δ = 162.8, 161.6, 156.6, 144.8, 130.5, 113.8, 113.1, 103.4, 102.2, 80.4, 73.5, 71.0, 70.1, 62.6, 62.2, 45.0, 27.8 ppm. $M_{n,SEC}$ = 3,100 Da, $Đ$ = 1.5. T_g = 4 °C.

Synthesis of p(CumGE-*stat*-ECH-*stat*-*t*BGE) 7. The experimental procedure is identical to that of p(CumGE-*stat*-ECH-*stat*-*t*BGE) **4** described in the main text. Polymer **7** was synthesized using p(ECH-*stat*-*t*BGE) **3** (1.53 g, 12.2 mmol ECH, 1.0 eq. ECH), K_2CO_3 (5.06 g, 36.6 mmol, 3.0 eq.) and 7-hydroxycoumarin (2.18 g, 13.4 mmol, 1.1 eq.). Yield: 0.76 g. 26 mol% CumGE, 54 mol% ECH, 19 mol% *t*BGE. ^{1}H-NMR (CD_3CN): δ = 7.74 (m, 2H$^{10,10'}$), 7.43 (d, 2H$^{8,8'}$), 6.83 (m, 4H$^{7,7',9,9'}$), 6.15 (d, 2H$^{11,11'}$), 4.06 - 3.37 (m, 15H$^{1\text{-}4,6}$), 1.13 (s, 9H^5) ppm. ^{13}C-NMR (CD_3CN): δ = 162.8, 161.5, 156.7, 144.6, 130.5, 130.2, 113.8, 113.5, 102.3, 79.7, 73.5, 70.8, 70.0, 62.3, 61.5, 45.0, 27.7 ppm. $M_{n,SEC}$ = 2,900 Da, $Đ$ = 1.4. T_g = 8 °C.

Synthesis of p(CumGE-*stat*-ECH-*stat*-*t*BGE) 8. The experimental procedure is identical to that of p(CumGE-*stat*-ECH-*stat*-*t*BGE) **4** described in the main text. Polymer **8** was synthesized using p(ECH-*stat*-*t*BGE) **3** (1.56 g, 12.4 mmol ECH, 1.0 eq. ECH), K_2CO_3 (5.16 g, 37.2 mmol, 3.0 eq.) and 7-hydroxycoumarin (4.45 g, 27.4 mmol, 2.2 eq.). Yield: 1.68 g. 43 mol% CumGE, 37 mol% ECH, 20 mol% *t*BGE. ^{1}H-NMR (CD_3CN): δ = 7.65 (m, 2H$^{10,10'}$), 7.35 (d, 2H$^{8,8'}$), 6.74 (m, 4H$^{7,7',9,9'}$), 6.11 (d, 2H$^{11,11'}$), 4.09 - 3.34 (m, 15H$^{1\text{-}4,6}$), 1.10 (s, 9H^5) ppm. ^{13}C-NMR (CD_3CN): δ = 162.7, 161.5, 156.4, 144.6, 130.5, 114.9, 113.7, 103.4, 102.2, 79.8, 73.5, 70.0, 62.3, 44.9, 27.7 ppm. $M_{n,SEC}$ = 3,100 Da, $Đ$ = 1.4. T_g = 24 °C.

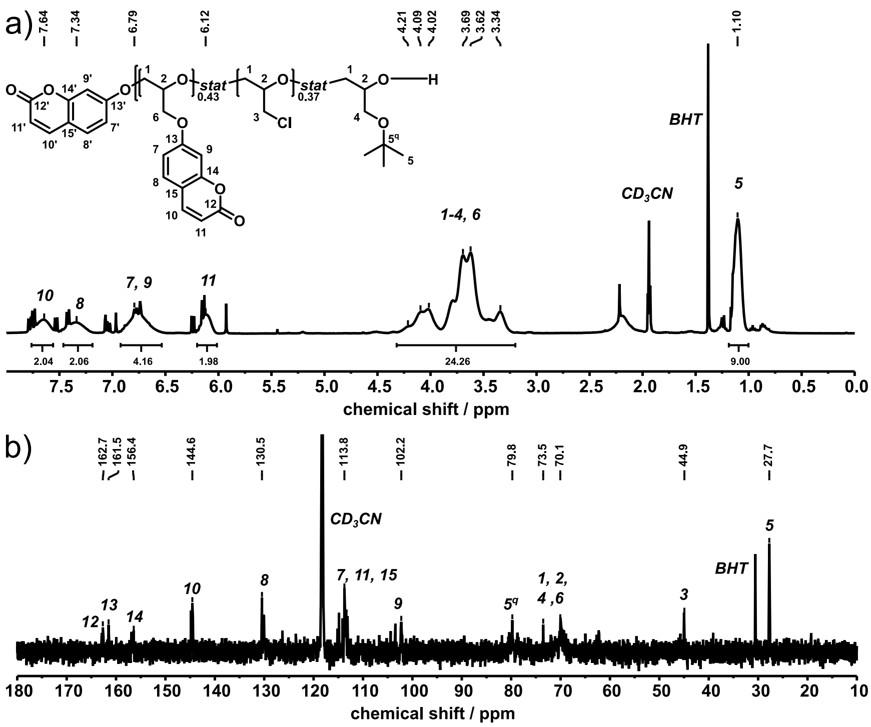

Figure S2. a) ^{1}H- and b) ^{13}C-NMR spectra of p(CumGE-*stat*-ECH-*stat*-*t*BGE) **8**, measured in CD_3CN.

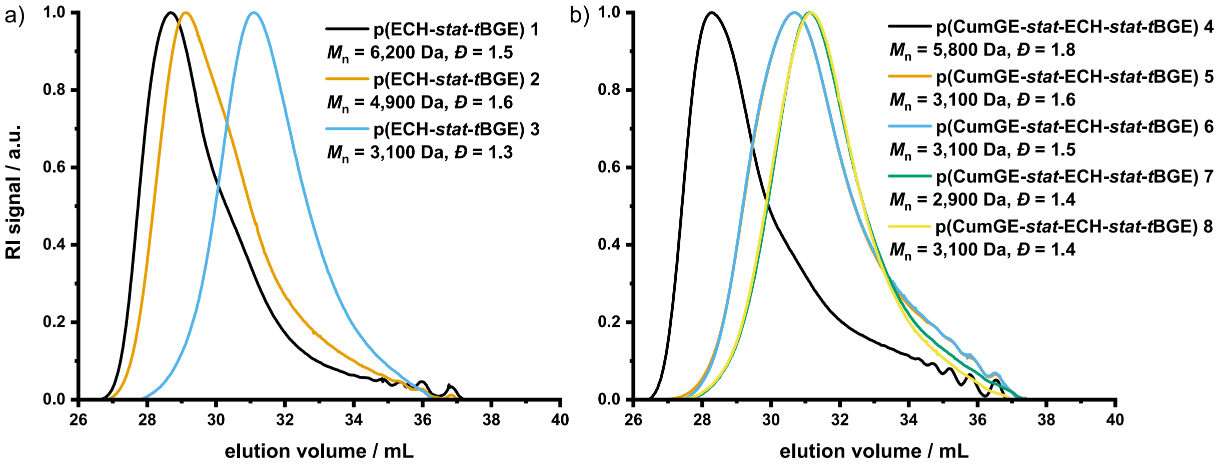

Figure S3. SEC elugrams of a) p(ECH-*stat*-*t*BGE) **1**–**3** and b) p(CumGE-*stat*-ECH-*stat*-*t*BGE) **5**–**8**, measured in THF.

Calculation of Monomer Ratios in p(ECH-*stat*-*t*BGE) 1-3 and p(CumGE-*stat*-ECH-*stat*-*t*BGE) 4-8

To calculate the molar ratio between ECH and *t*BGE repeating units in **1-3**, ^{1}H-NMR spectra were used. Each spectrum was normalized to the *t*Bu group of *t*BGE at $\delta = 1.16$ ppm. The fraction of a monomer x (r_x) can be obtained from the integral of a peak $\int H^{\#}$ unique to the monomer divided by the number of contributing nuclei N:

$$r_x = \frac{1}{N}\int \mathrm{H}^{\#}$$

The calculation is demonstrated using the ^{1}H-NMR spectrum of polymer **2** (Figure S2). The unique peak of H^5 of *t*BGE at $\delta = 1.16$ ppm is used to determine $r_{t\mathrm{BGE}}$:

$$r_{t\mathrm{BGE}} = \frac{1}{9}\int \mathrm{H}^5 = \frac{1}{9} \cdot 9 = 1$$

No distinct unique peak of ECH is available, as the RCH_2Cl signal H^3 overlaps with the signals of the polymer backbone. Thus, the integral of $H^{1\text{-}4}$ has to be corrected by 5 protons, corresponding to the *t*BGE repeating unit. r_{ECH} is calculated using:

$$r_{ECH} = \frac{1}{5}\int \mathrm{H}^{1-4} - 5 = \frac{1}{5} \cdot (12.56 - 5) = 1.5$$

The ratio of ECH and *t*BGE in polymer **2** is 1.5 : 1 (60 mol% ECH, 40 mol% *t*BGE).

The same procedure is used to obtain the molar ratios of CumGE, ECH and *t*BGE in polymers **4-8** and will be shown at the example of polymer **5** (^{1}H-NMR spectrum: Figure S4). Again, because the spectrum was normalized to the 9 protons of *t*BGE's *tert*-butyl group H^5, $r_{t\mathrm{BGE}} = 1$. Peak H^{11}, which is unique to a single proton of a coumarin-functionalized repeating unit, is used to calculate r_{CumGE}:

$$r_{\mathrm{CumGE}} = \frac{1}{1}\int \mathrm{H}^{11} = \frac{1}{1} \cdot 0.65 = 0.65$$

Again, no distinct ECH peak can be used for direct comparison of the monomers. Due to the additional presence of CumGE repeating units, polymer backbone signals $H^{1\text{-}4,6}$ does not

only need to be corrected by the 5 protons of one *t*BGE but also by 5 protons of 0.65 CumGE repeating units. r_{ECH} is obtained by:

$$r_{\text{ECH}} = \frac{1}{5}\int \text{H}^{1-4,6} - 5 - 5 \cdot 0.65 = \frac{1}{5} \cdot (12.94 - 5 - 3.25) = 0.94$$

Thus, the ratio of CumGE, ECH and *t*BGE in polymer **5** is 0.65 : 0.94 : 1 (25 mol% CumGE, 36 mol% ECH, 39 mol% *t*BGE).

Calculation of the Molecular Weight of p(CumGE-*stat*-ECH-*stat*-*t*BGE) 4 by NMR

The number-average molecular weight $M_{\text{n,NMR}}$ of polymer **4** can be calculated, because protons H^8 of side group (SG) and end group (EG) coumarin moieties give distinct signals at $d = 7.48$ and 7.42 ppm, respectively, in the ^{1}H-NMR spectrum (Figure S5). With the following equations, the degree of polymerization DP_{x} of each constituent monomer x can be calculated using the same unique ^{1}H-NMR signals previously used for the calculation of monomer ratios:

$$DP_{t\text{BGE}} = \frac{\frac{1}{9}\int \text{H}^5}{\frac{1}{1}\int \text{H}^{8,\text{EG}}} = \frac{\frac{1}{9}\cdot 9}{0.04} = 25$$

$$DP_{\text{CumGE}} = \frac{\frac{1}{1}\int \text{H}^{8,\text{SG}}}{\frac{1}{1}\int \text{H}^{8,\text{EG}}} = \frac{0.05}{0.04} \approx 1$$

$$DP_{\text{ECH}} = \frac{\frac{1}{5}(\int \text{H}^{1-4,6} - 5 \cdot \frac{1}{1}\int \text{H}^{8,\text{SG}} - 5 \cdot \frac{1}{1}\int \text{H}^{8,\text{EG}} - 5 \cdot \frac{1}{9}\int \text{H}^5)}{\frac{1}{1}\int \text{H}^{8,\text{EG}}} = \frac{0.16}{0.04} = 4$$

The combined degree of polymerization for all monomers is 30, while a chain length of 25 was targeted in the initial polymerization. This result is within the general margin of error for ^{1}H-NMR measurements. Lastly, $M_{\text{n,NMR}}$ can be calculated by:

$$M_{\text{n,NMR}} = DP_{t\text{BGE}} \cdot 130.18\ \text{g} \cdot \text{mol}^{-1} + DP_{\text{ECH}} \cdot 92.53\ \text{g} \cdot \text{mol}^{-1} + DP_{\text{CumGE}} \cdot 218.21\ \text{g} \cdot \text{mol}^{-1}$$

$$M_{n,NMR} = 25 \cdot 130.18\ \mathrm{g \cdot mol^{-1}} + 4 \cdot 92.53\ \mathrm{g \cdot mol^{-1}} + 1 \cdot 218.21\ \mathrm{g \cdot mol^{-1}} \approx 3{,}800\ \mathrm{g \cdot mol^{-1}}$$

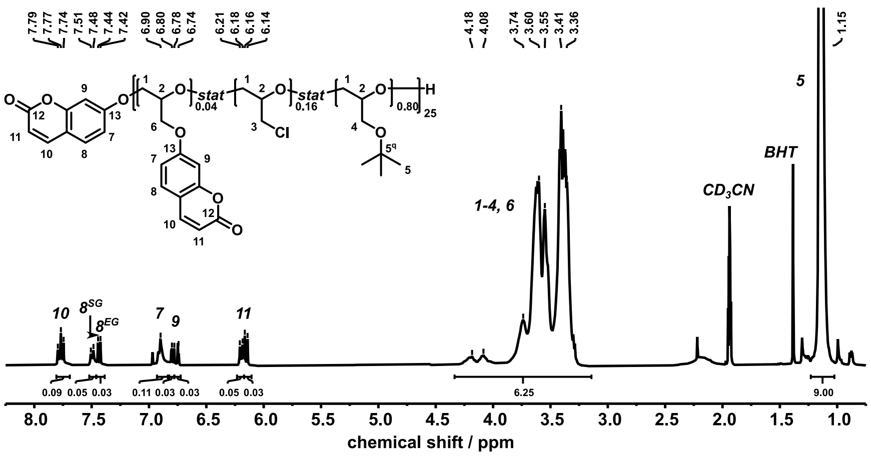

Figure S4. ^{1}H-NMR spectrum of p(CumGE-*stat*-ECH-*stat*-*t*BGE) **4**, used for the calculation of the molecular weight $M_{n,NMR}$ by end group analysis. Measured in CD_3CN.

Synthesis of 2,2-di-pyrazol-1-yl-ethanamine. The amine functionalized scorpionate ligand was synthesized following the procedure of Reger et al..[4] 2,2-Di-pyrazol-1-yl-ethanamine was obtained after a three step synthesis (Scheme S1) starting from 1,8-naphthalic anhydride and 1-aminoacetaldehyde dimethylacetal as brown solid. Yield: >90%. ^{1}H-NMR (DMSO-d6) δ = 7.92 (d, J = 2.4 Hz, 2H), 7.50 (d, J = 1.6 Hz, 2H), 6.48 (t, J = 7.2 Hz, 1H), 6.32 - 6.22 (m, 2H), 3.54 (d, J = 7.2 Hz, 2H) ppm.

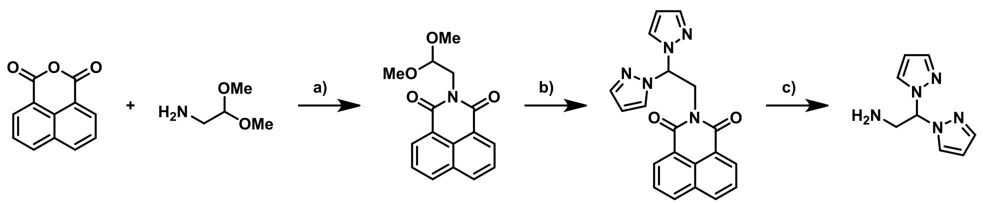

Scheme S1. Synthesis of 2,2-Di-pyrazol-1-yl-ethanamine following the procedure of Reger et al.: a) EtOH, reflux, 4h; b) 1H-pyrazole, p-TsOH, 200 - 220 °C; c) $N_2H_4 \cdot H_2O$, toluene, reflux, 12 h.[4]

Synthesis of MG II-pz. Experimental data can be found in the main text. ^{1}H-NMR (DMSO-d6) δ= 7.99 (d, 2H, H^2), 7.57 (d, 2H, H^4), 6.93 (m, 1H, H^7), 6.34 (m, 2H, H^3), 6.25 (m, 2H, $H^{5,6}$), 4.5 - 3.0 (m, cyclobutane and backbone), 4.05 (m, 2H, H^1), 1.10 (s, 9H, *t*BGE) ppm.

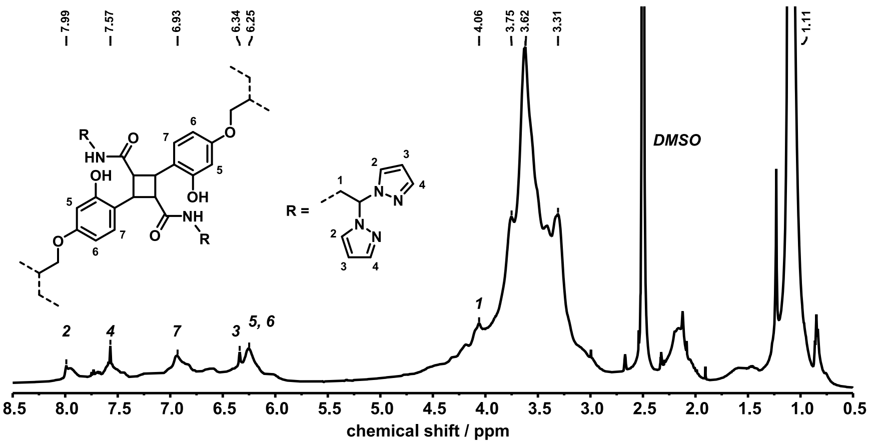

Figure S5. ^{1}H-NMR spectrum of MG **II**-pz, measured in DMSO-d6.

Optimization of Microgel Synthesis

Microgel synthesis conditions were optimized by comparing the crosslinking reaction of p(CumGE-*stat*-ECH-*stat*-*t*BGE) **4** in abscence or presence of two different photosensitizers (benzophenone or 2,2-dimethoxy-2-phenylacetophenone – DMPA) in the crosslinking procedure. The crosslinking reaction was performed in a miniemulsion, containing **4** (0.25 g), hexadecane (0.02 g) and photosensitizer (0.05 mmol) in toluene (0.88 g) as organic phase and SDS (0.002 g) dissolved in water (4 g) as aqueous phase. After combining both phases, the miniemulsion was prepared using a Branson Ultrasonifier 450 for 15 min (output control 3, duty cycle 50%). The mixture was irradiated for 3 h at λ = 365 nm under vigorous mechanical stirring. After the reaction, a small sample of the reaction mixture was dried, redissolved in MeCN and investigated using UV-Vis spectrometry (Figure S8).

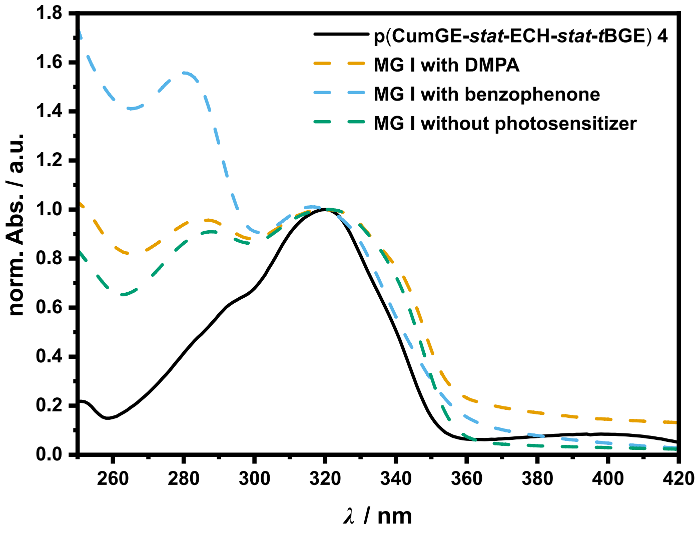

Figure S6. UV-Vis spectra, measured in MeCN, of a) linear p(CumGE-*stat*-ECH-*stat*-*t*BGE) **4**, and microgels obtained after photochemical crosslinking in miniemulsion b) in abscence of photosensitizer, c) in presence of benzophenone and d) in presence of DMPA.

Confocal Image of MG II

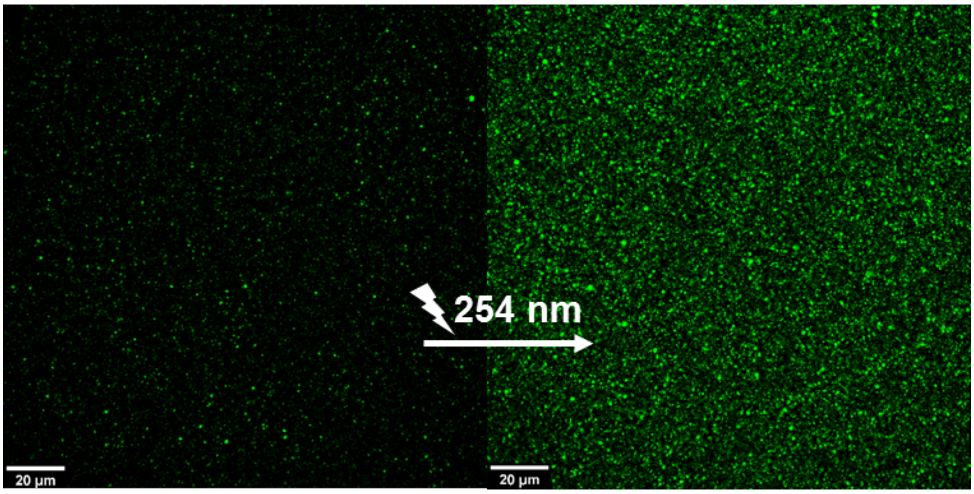

Figure S7. Confocal microscopy image of dispersed MG **II** before (left) and after (right) irradiation at 254 nm.

IR Spectra of MG I at Different pH

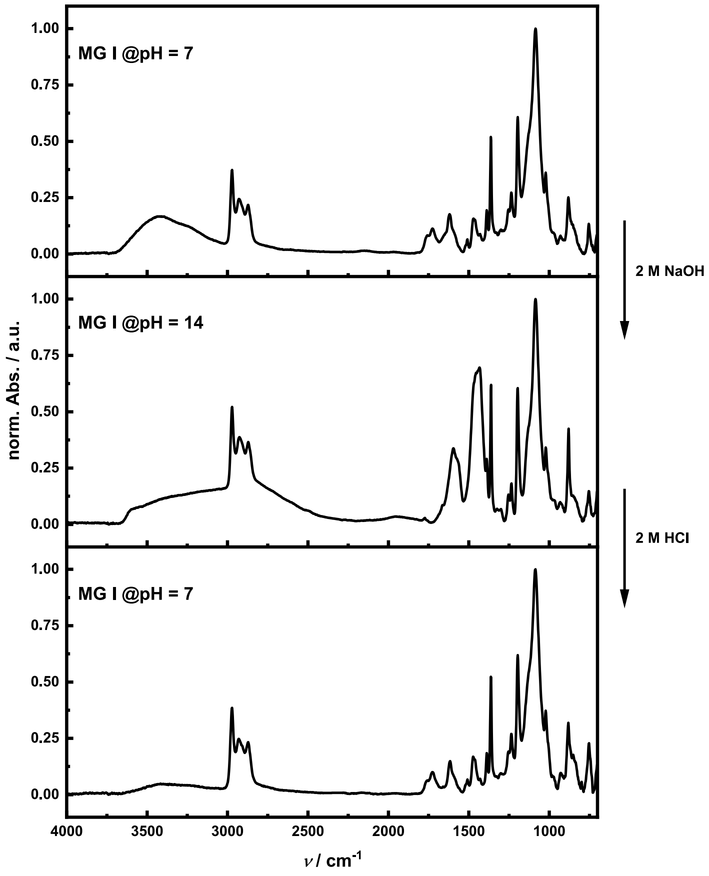

Figure S8. FTIR spectra of MG **I** at pH 7 (top), after raising the pH to 14 through addition of some 2 M NaOH (center) and after subsequent neutralization to pH 7 using some 2 M HCl (bottom).

Differential Scanning Calorimetry

Table S1. Glass transition temperatures for the polymers **1**-**8** and microgel **I**-**IV** analyzed by differential scanning calorimetry (DSC).

**polymer**	T_g / °C	**polymer**	T_g / °C	**microgel**	T_g / °C
**1**	-36	**4**	-15	**I**	-6
**2**	-29	**5**	2	**II**	12
**3**	-33	**6**	4	**III**	29
		**7**	8	**IV**	21
		**8**	24	**-**	-

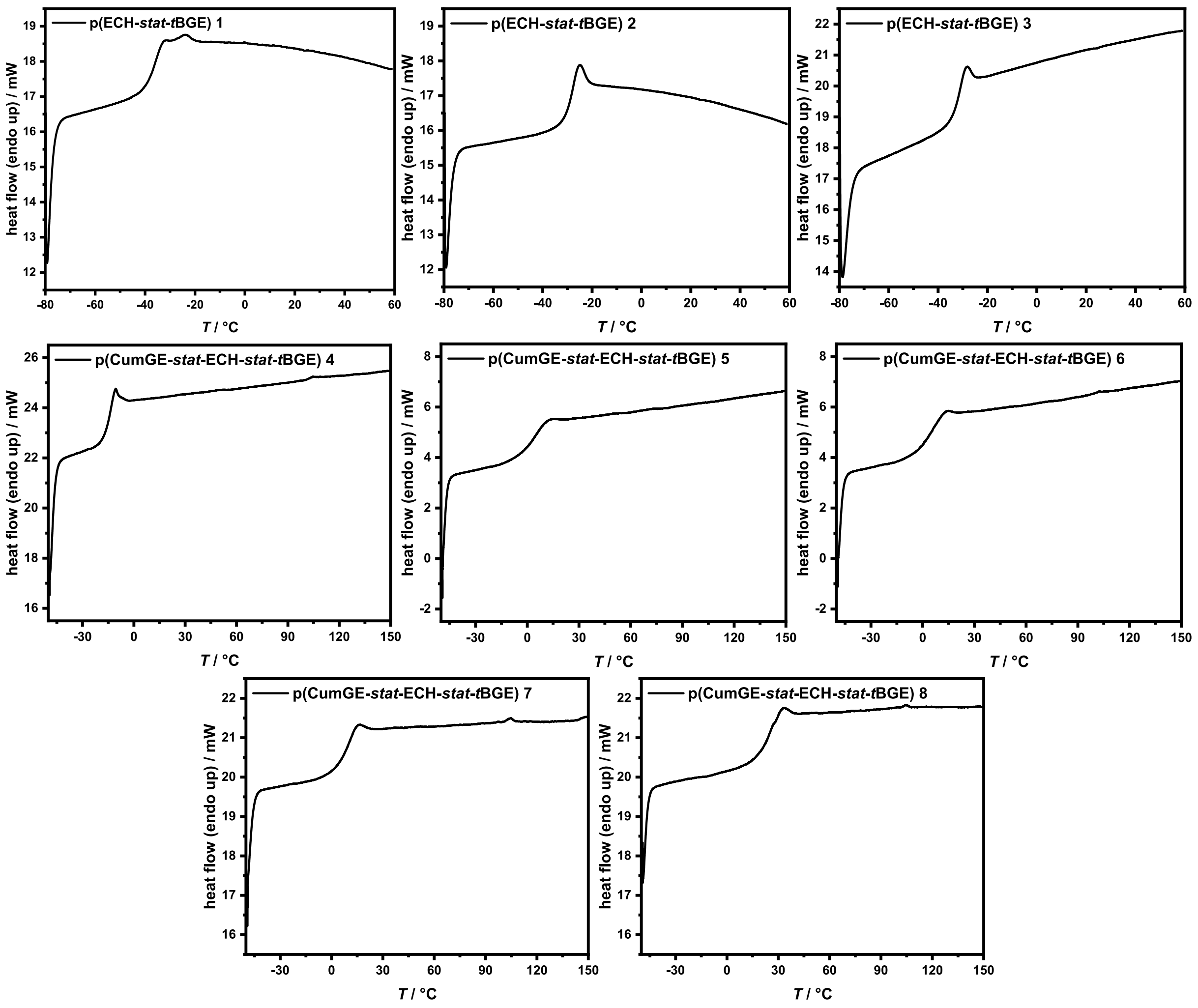

Figure S9. Second heating curves ($\Delta T = 20$ K·min^{-1}) of DSC measurements of polymer **1**-**8**, used for the calculation of glass transition temperatures T_g.

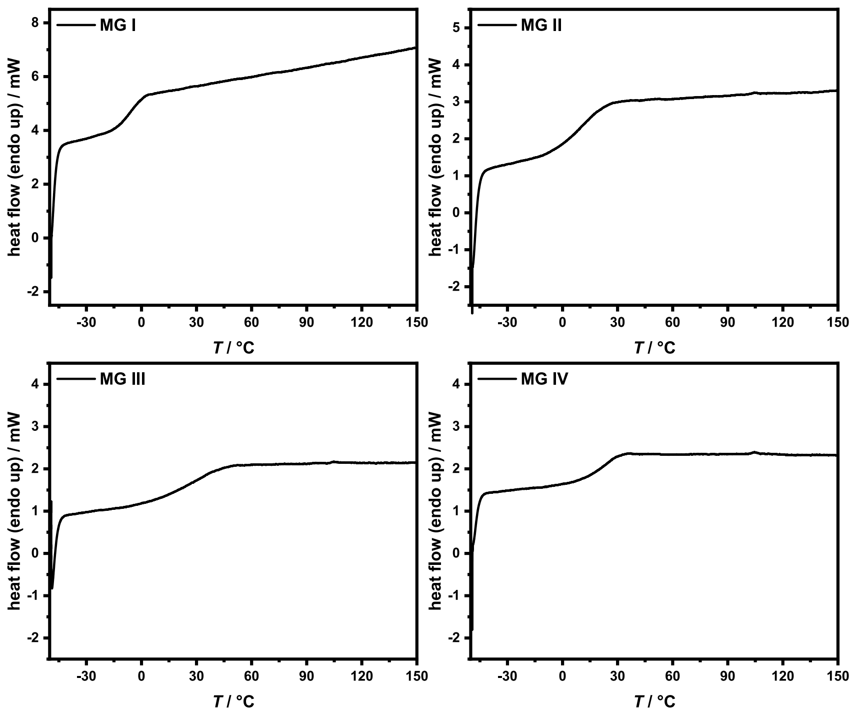

Figure S10. Second heating curves (ΔT = 20 K·min^{-1}) of DSC measurements of MG **I-IV**, used for the calculation of glass transition temperatures T_g.

X-ray Photoelectron Spectroscopy

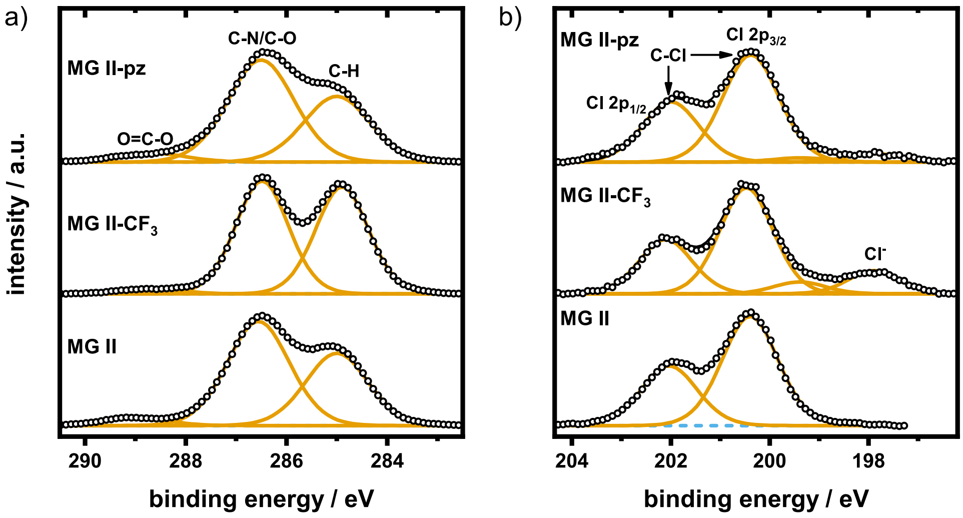

Figure S11. a) C 1s and b) Cl 2p XP spectra of microgel MG **II** (bottom) and the functionalized microgels MG **II**-CF_3 (center) and MG **II**-pz (top), respectively.

Calculation of Light Penetration Depth During Photocrosslinking

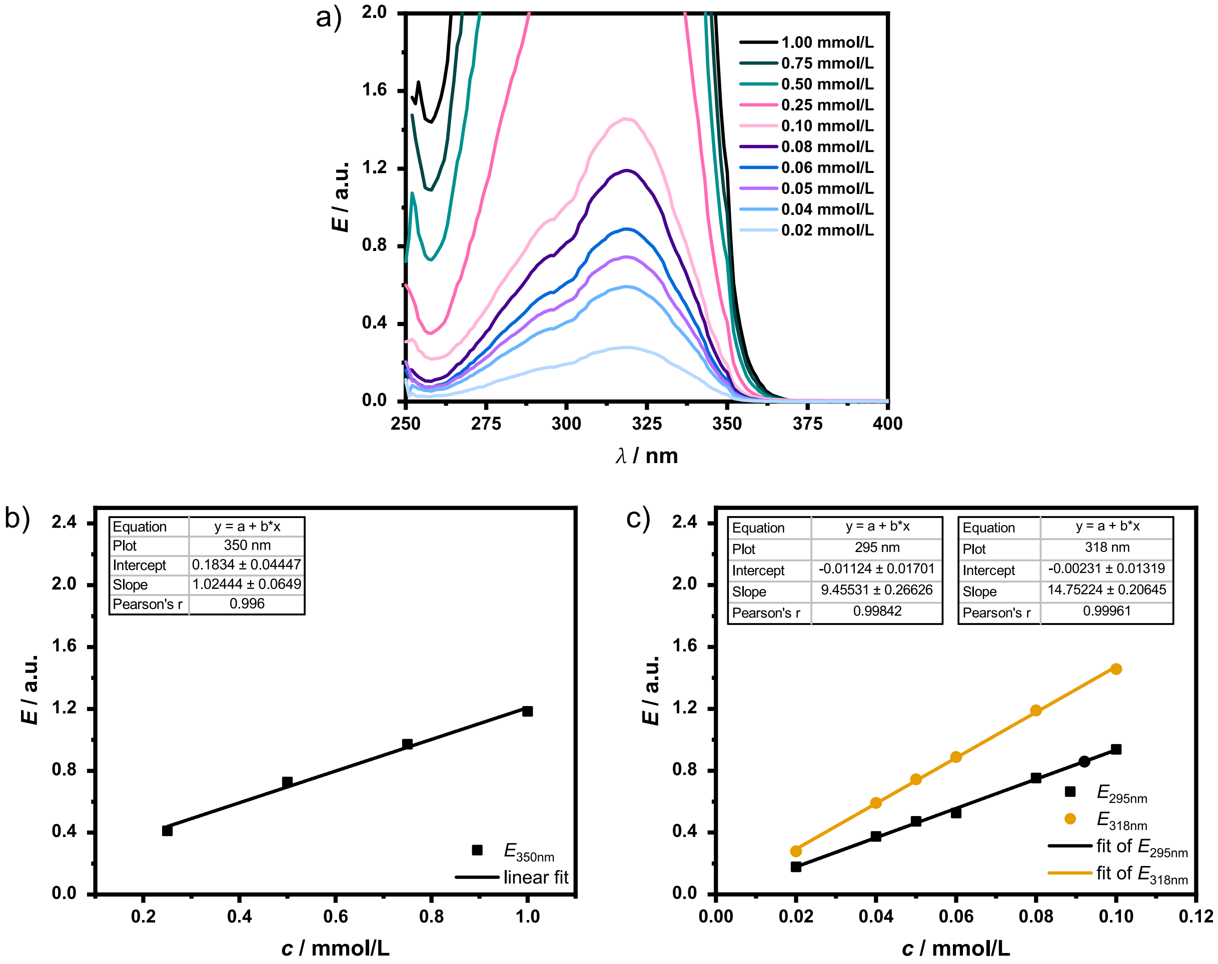

Figure S12. a) Stacked UV-Vis spectra of 7-methoxycoumarin, measured in acetonitrile. Determination of attenuation coefficient ε through linear regression of extinction E at b) $\lambda = 350$ nm ($\varepsilon_{350\text{nm}} = 1{,}020$ L·mol^{-1}·cm^{-1}) and c) 318 nm ($\varepsilon_{318\text{nm}} = 14{,}750$ L·mol^{-1}·cm^{-1}) and 295 nm ($\varepsilon_{295\text{nm}} = 9{,}450$ L·mol^{-1}·cm^{-1}).

UV-Vis absorption spectra of 7-methoxycoumarin in acetonitrile, serving as a small molecule model compound for polymerized coumaryl glycidyl ether repeating units, were recorded to determine the attenuation coefficients at $\lambda = 295$, 318 and 350 nm (Figure S12). To estimate the attenuation of light during photocrosslinking of **5**, the concentration of coumarin moieties in the toluene phase of the reaction emulsion must be calculated:

$$\overline{M}_5 = r_{\mathrm{CumGE}} \cdot M_{\mathrm{CumGE}} + r_{t\mathrm{BGE}} \cdot M_{t\mathrm{BGE}} + r_{\mathrm{ECH}} \cdot M_{\mathrm{ECH}}$$

$$\overline{M}_5 = (0.25 \cdot 218.21 + 0.39 \cdot 130.19 + 0.36 \cdot 92.52) \frac{\mathrm{g}}{\mathrm{mol}} = 138.63 \frac{\mathrm{g}}{\mathrm{mol}}$$

$$n_{\mathrm{CumGE}} = r_{\mathrm{CumGE}} \cdot \frac{m_5}{\overline{M}_5} = 0.25 \cdot \frac{250\ \mathrm{mg}}{138.63 \frac{\mathrm{g}}{\mathrm{mol}}} = 0.451\ \mathrm{mmol}$$

$$c_{\mathrm{CumGE}} = \frac{n_{\mathrm{CumGE}}}{V_{\mathrm{PhMe}}} = \frac{0.451\ \mathrm{mmol}}{1.0\ \mathrm{mL}} = 0.451\ \frac{\mathrm{mol}}{\mathrm{L}}$$

The largest nanocapsule of **II** measured via AFM had a diameter of 400 nm (Figure 8e). Because the emulsion was stirred during crosslinking, light must penetrate at least 200 nm into the toluene phase to ensure crosslinking of all coumarin moieties if no surfactant-like templating took place. Using Beer-Lambert's law and the previously determined attenuation coefficients, the transmittance T at $\lambda = 318$ and 350 nm can be obtained from the following equations:

$$E = \varepsilon \cdot c_{\mathrm{CumGE}} \cdot d$$

$$T = 10^{2-E}$$

$$E_{350\mathrm{nm}} = 1{,}020 \frac{\mathrm{L}}{\mathrm{mol} \cdot \mathrm{cm}} \cdot 0.451\ \frac{\mathrm{mol}}{\mathrm{L}} \cdot 2 \cdot 10^{-5}\ \mathrm{cm} = 9.20 \cdot 10^{-3}$$

$$T_{350\mathrm{nm}} = 10^{2-9.20 \cdot 10^{-3}} = 0.979$$

$$E_{318\mathrm{nm}} = 14{,}750 \frac{\mathrm{L}}{\mathrm{mol} \cdot \mathrm{cm}} \cdot 0.451\ \frac{\mathrm{mol}}{\mathrm{L}} \cdot 2 \cdot 10^{-5}\ \mathrm{cm} = 0.133$$

$$T_{318\mathrm{nm}} = 10^{2-0.133} = 0.736$$

References

1. Parry, K. L.; Shard, A. G.; Short, R. D.; White, R. G.; Whittle, J. D.; Wright, A., ARXPS characterisation of plasma polymerised surface chemical gradients. *Surf. Interface Anal.* **2006,** *38* (11), 1497-1504.
2. Scofield, J. H., Hartree-Slater subshell photoionization cross-sections at 1254 and 1487 eV. *J. Electron. Spectros. Relat. Phenomena* **1976,** *8* (2), 129-137.
3. Tanuma, S.; Powell, C. J.; Penn, D. R., Calculations of electron inelastic mean free paths. V. Data for 14 organic compounds over the 50-2000 eV range. *Surf. Interface Anal.* **1994,** *21* (3), 165-176.
4. Reger, D. L.; Semeniuc, R. F.; Gardinier, J. R.; O'Neal, J.; Reinecke, B.; Smith, M. D., New N,N,N-heteroscorpionates based on 2,2'-bis(pyrazolyl)ethanamine and its derivatives. Ligands designed for probing supramolecular interactions. *Inorg. Chem.* **2006,** *45* (11), 4337-4339.
